# Do Not Let the Guard Down on Preventable Behavioral Risk Factors

**DOI:** 10.2188/jea.JE20250037

**Published:** 2025-11-05

**Authors:** Chiara Stival, Anna Odone, Alessandra Lugo, Piet A. van den Brandt, Silvio Garattini, Silvano Gallus

**Affiliations:** 1 Department of Medical Epidemiology, Istituto di Ricerche Farmacologiche Mario Negri IRCCS, Milan, Italy; 2 Department of Public Health, Experimental and Forensic Medicine, University of Pavia, Pavia, Italy; 3 Medical Direction, Fondazione IRCCS Policlinico San Matteo, Pavia, Italy; 4 Maastricht University Medical Centre, GROW- School for Oncology and Developmental Biology, Department of Epidemiology, Maastricht, the Netherlands; 5 Maastricht University Medical Centre, CAPHRI- School for Public Health and Primary Care, Department of Epidemiology, Maastricht, the Netherlands; 6 Istituto di Ricerche Farmacologiche Mario Negri IRCCS, Milan, Italy

**Keywords:** primary prevention, risk factors, noncommunicable diseases, bibliometric analysis, literature review

## Abstract

**Background:**

Preventable behavioral risk factors account for approximately one third of mortality, morbidity, and disability worldwide. This study aims to quantify the interest in behavioral risk factors within major medical journals in 2022 and to derive trends over the past 30 years in the entire medical literature.

**Methods:**

We analyzed the proportion of publications dealing with tobacco smoking, alcohol drinking, use of illicit drugs, excess body weight and physical activity among all the 1,128 articles published in the Journal of the American Medical Association, the British Medical Journal, the Lancet, and New England Journal of Medicine in 2022. A joinpoint analysis was conducted running in PubMed/MEDLINE specific search strings to evaluate trends over the last 30 years in the four journals and in the whole medical literature.

**Results:**

In 2022, of all publications from the four considered medical journals, 2.8% dealt with tobacco smoking, 1.6% alcohol drinking, 1.1% use of illicit drugs, 3.8% excess body weight, 2.7% physical activity and 8.0% dealt with any behaviors. The joinpoint analysis on the whole medical literature showed that papers on modifiable risk factors significantly increased from 3.9% in 1993 to 6.2% in 2014 (annual percent change [APC]: between +1.83% and +4.09%), and subsequently decreased between 2014 and 2019 (APC −0.31%), with an acceleration thereafter (APC −2.41% in 2019–2022).

**Conclusion:**

For the first time we quantified the volume of medical research focused on preventable behavioral risk factors. This appears to be limited and declining over the last decade. Research on primary prevention should be a priority to face the emergence of associated non-communicable diseases globally.

## INTRODUCTION

Despite the massive increase in global life-expectancy in recent decades, millions of years lost due to disability, morbidity, and premature mortality are reported every year.^1^^,^^2^ Non-communicable diseases (NCD) are chronic diseases that are not caused by infectious agents and cannot be transmitted from person to person. They typically develop over a long period and result from a combination of genetic, environmental, and behavioral risk factors. Indeed, preventable behavioral risk factors, such as tobacco smoking, alcohol drinking, excess body weight, and physical inactivity, all increase the risk of total mortality for NCDs and injuries.^2^^–^^4^ The Global Burden of Diseases (GBD) showed how these preventable behavioral risk factors are among the main causes of disability and premature mortality worldwide,^2^ contributing up to 60% of premature deaths.^5^ According to latest GBD estimates (2021), behavioral risk factors explain approximately one third of global mortality and around one fourth of total years lost for morbidity, disability, and premature mortality.^2^ These data reveal that, although a healthy lifestyle has repeatedly been shown to improve life expectancy and well-being,^3^^,^^4^ the proportion of the population exposed to unhealthy lifestyles remains high.^6^

An important way to control NCDs is to set preventive strategies for governments and other stakeholders to promote a healthy lifestyle to reduce NCDs’ behavioral risk factors.^7^ Despite the fact that preventive measures of unhealthy behaviors have proven to be effective in controlling the onset of diseases^8^^,^^9^ and reducing their economic burden,^10^^,^^11^ a substantial proportion of healthcare resources and research funds are still allocated to disease management, rather than to preventive activities. Data from 2010–2012, revealed that the United States National Institute of Health (NIH) devoted only less than 10% of its budget to financially support projects on disease prevention or health promotion.^12^ In particular, of the total funds allocated to interventions for prevention, 6% were on tobacco; 3% were on alcohol drinking; 2% were on illicit drugs; 41% were on diet, excess body weight, and physical activity; 14% were on mental health; 12% were on a combination of such risk factors; and the remaining 22% were on social determinants of health.^12^ A more recent study showed that, in the fiscal years 2012–2017, only one sixth of projects funded by NIH were used for primary and secondary prevention research in the United States.^13^ An update of the study revealed that, in the fiscal year 2019, 21% of the projects supported by NIH were focused on primary and secondary prevention research.^14^

The scarcity of funds for prevention may be reflected in shifting research priorities and funding patterns, rather than in a diminished recognition of the critical role of prevention in public health, and may also contribute to a general lack of interest in this topic within the medical literature. Accordingly, it is expected that the largest share of medical research is focused on the development of disease treatments. A recent systematic review of the medical literature found that only 14% of European cancer research papers had a focus on primary or secondary prevention research.^15^ To our knowledge, no study has yet analyzed the interest in the main preventable behavioral risk factors in the medical literature.

In order to assess the attention paid to the prevention of behavioral risk factors in medical research, we manually checked and coded all the publications from the major general medicine journals for the most recent complete year, 2022. Our aim was to quantify the proportion of studies focused on tobacco smoking, alcohol drinking, use of illicit drugs, excess body weight and physical activity, key targets for primary prevention efforts, as largely avoidable and modifiable through individual lifestyle choices. We also provide temporal trends of the proportion of articles on behavioral risk factors in the main medical journals and in the whole medical literature over the last 30 years.

## METHODS

### Analysis of individual articles from four main journals of general medicine, 2022

We searched all articles published in 2022 in the four major internationally recognized journals of general medicine (Journal of the American Medical Association [JAMA], the British Medical Journal [BMJ], The Lancet, and the New England Journal of Medicine [NEJM]). In order to more easily access information from all papers, we decided to consider only those with abstract available. Overall, 1,128 papers were obtained from the selection. The title and abstract of all the papers was manually screened and articles were coded according to their study type (ie, interventional, observational, review, and other design); study design; the country where the studies were conducted, whenever possible; and the topic of interest (ie, cardiovascular disease [CVD], coronavirus disease 2019 [COVID-19], mental health, cancer, digestive system and metabolic diseases, orthopedic diseases, female reproductive system diseases, respiratory diseases, sexually transmitted diseases [STDs], and other diseases). The publications were categorized according to their focus on specific preventable behavioral risk factors, including tobacco smoking, alcohol drinking, use of illicit drugs, excess body weight, and physical activity. To further characterize the publications and better identify those dedicated to interventions aimed at decreasing unhealthy behaviors, the role of such behaviors was determined within each article and exhaustively classified as follows: i) “behavior as outcome”, if the research dealt with interventions to modify the behavior; ii) “behavior as exposure” if it dealt with the effect of such behavior on specific outcomes; iii) “behavior as burden” if it dealt with the frequency or burden of the behavior on health; and iv) “behavior as recommendation” if it dealt with recommendations about such behavior.

### Time trend analysis (PubMed/MEDLINE, 1993–2022)

In order to evaluate trends in the last 30 years (calendar period 1993–2022) of the proportion of publications dealing with preventable behavioral risk factors we ran in PubMed/MEDLINE search strings—based on Medical Subject Headings (MeSH) terms and keywords—built to identify publications related to tobacco smoking, alcohol drinking, illicit drugs, excess body weight, and physical activity (details on the search strings are reported in eTable 1).

The trend analysis was performed on the annual proportion of papers dealing with any or specific behaviors for the period 1993–2022. Two sets of data were considered: i) the four selected main general medicine journals combined and ii) the whole scientific literature (ie, considering all the journals indexed in PubMed/MEDLINE with abstract available).

### Statistical analysis

Within the 2022 publications of the four considered journals, the proportion of those dealing with preventable behavioral risk factors—either as outcome, exposure, their burden, or their recommendations—was provided overall and according to selected article characteristics. In order to identify those most closely related to the prevention of specific behaviors, we also provided the proportion of publications dealing with the behavior as exposure, its burden and its recommendations, only.

For each article from the four major journals, a dichotomous variable was created, coded as 0 if the article did not deal with preventable behavioral risk factors and 1 if it did. To explore the correlates of articles addressing preventable behavioral risk factors, we used unconditional multiple logistic regression models to estimate the adjusted odds ratios (aORs) and their 95% confidence intervals (CIs), adjusting for journal, study type, country, and article topic.

The aOR and 95% confidence intervals (CI) of papers dealing with preventable behavioral risk factors according to selected article characteristics were derived using unconditional multiple logistic regression models after adjustment for journal, study type, country and article topic.

In order to provide information on the accuracy of our search strings used in PubMed/MEDLINE to identify papers on specific behavioral risk factors, we analyzed the 1,128 papers published in 2022 in JAMA, BMJ, the Lancet, and NEJM, comparing the proportion of papers on prevention identified by the strings with that identified by the reviewer (used as gold standard).

The trends over the last three decades in proportions of publications focused on the considered behavioral risk factors in the four medical journals and in the whole PubMed/MEDLINE were analyzed using joinpoints regression models, allowing for up to six joinpoints. For each trend, the Joinpoint program estimates six segmented linear regression models (0–6) and, among them, selects the best one based on the weighted Bayesian Information Criteria (BIC).^16^ Within each segment line the average annual percent change (APC) synthetically explains the sign and the intensity of the variation of the measure of interest.^16^ Trends of proportions of publications on preventable behavioral risk factors for each of the four journals were also plotted.

All statistical analyses were performed using the software SAS 9.4 (SAS Institute, Cary, NC, USA), the Joinpoint Regression Program version 5.0.2 (Statistical Methodology and Applications Branch, Surveillance Research Program, National Cancer Institute, Bethesda, MD, USA) and R (R Foundation For Statistical Computing, Vienna, Austria).

## RESULTS

### Analysis of individual articles

Of the 1,128 papers published in the four main journals of general medicine in 2022 (eTable 2), 409 (36.3%) were published in JAMA, 239 (21.2%) in BMJ, 253 (22.4%) in the Lancet, and 227 (20.1%) in NEJM (eTable 3). Overall, interventional studies (34.0%) were the most frequent category, followed by observational studies (28.7%) and reviews (18.1%). The remaining 19.2% were other types of studies, including in vivo and in vitro study types. Overall, 43.3% of the studies were conducted in the United Kingdom or North America. The main topic of interest was COVID-19 (16.8%), followed by CVD (15.3%), mental or brain related diseases (11.3%), cancer (8.8%), and digestive system diseases (8.8%).

Overall, 90 papers (8.0%) dealt with preventable behavioral risk factors. In particular, 2.8% were on tobacco smoking,1.6% were on alcohol drinking, 1.1% were on use of illicit drugs, 3.8% were on excess body weight, and 2.7% were on physical activity (Table 1 and eTable 4). Multivariate analysis revealed that, compared with articles from JAMA, papers from BMJ more frequently dealt with preventable behavioral risk factors (aOR 1.96; 95% CI, 1.00–3.85), whereas those from the Lancet were not statistically different. Publications from NEJM related less to preventable behavioral risk factors (aOR 0.36; 95% CI, 0.14–0.94) compared to those from JAMA. Overall, there was no statistically significant difference in the odds of papers dealing with preventable behavioral risk factors according to study type. Papers from North America dealt more frequently with preventable behavioral risk factors (aOR 2.78; 95% CI, 1.12–6.91), compared to those from the United Kingdom. In comparison with papers on CVDs, those on COVID-19 less frequently dealt with preventable risk factors (aOR 0.13; 95% CI, 0.03–0.60). In contrast, those on mental health (aOR 3.68; 95% CI, 1.73–7.83) and those on the digestive system and metabolic diseases (aOR 6.12; 95% CI, 2.85–13.16) more frequently dealt with preventable risk factors. When we considered only articles dealing with the preventable behavioral risk factor as an exposure, the burden of the behavior and its recommendation (thus excluding those considering the behavior as an outcome), 63 (5.6%) papers dealt with any behavior, 26 (2.3%) with smoking, 17 (1.5%) with alcohol, 8 (0.7%) with illicit drugs, 31 (2.7%) with excess body weight, and 23 (2.1%) with physical activity (eTable 5).

**Table 1.** Distribution of 1,128 papers published in JAMA, BMJ, the Lancet, and NEJM in 2022 by their focus on selected preventable behavioral risk factors (ie, smoking, alcohol drinking, use of illicit drugs, excess body weight, and physical activity) overall and according to selected characteristics

Characteristics of the included studies	Number	Any preventable behavioral risk factor	

		%	aOR^a^ (95% CI)
All	1,128	8.0	—
Journal			
JAMA	409	8.6	1.00^b^
BMJ	239	11.7	**1.96 (1.00**–**3.85)**
Lancet	253	7.9	1.20 (0.59–2.47)
NEJM	227	3.1	**0.36 (0.14**–**0.94)**
Study Type			
Interventional	384	6.5	1.00^b^
Observational	324	9.9	1.48 (0.76–2.86)
Review	204	12.3	1.98 (0.70–5.65)
Other^c^	216	3.7	0.63 (0.23–1.74)
Country			
UK^d^	136	6.6	1.00^b^
North America	352	10.8	**2.78 (1.12**–**6.91)**
European countries other than UK	90	5.6	1.30 (0.38–4.42)
Africa, Asia, Oceania and Other American countries	103	3.9	1.31 (0.35–4.92)
Multicontinent	174	6.3	2.40 (0.85–6.80)
Not applicable	273	8.4	0.95 (0.29–3.12)
Type of disease^e^			
CVD	173	6.9	1.00^b^
COVID-19	189	1.1	**0.13 (0.03–0.60)**
Mental health	128	21.1	**3.68 (1.73–7.83)**
Cancer	99	7.1	1.13 (0.42–3.06)
Digestive system and metabolic diseases	99	29.3	**6.12 (2.85–13.16)**
Orthopedic diseases	48	6.3	0.89 (0.24–3.38)
Female reproductive system diseases	52	5.8	0.81 (0.22–3.03)
Respiratory diseases	40	7.5	1.04 (0.27–4.01)
STD	56	1.8	0.30 (0.04–2.43)
Other	244	1.2	**0.17 (0.05–0.63)**

aOR, adjusted odds ratio; BMJ, British Medical Journal; CI, confidence interval; COVID-19, coronavirus disease 2019; CVD, cardiovascular disease; NEJM, New England Journal of Medicine; STD, sexually transmitted diseases and sexuality; UK, United Kingdom.

^a^Estimated by unconditional multiple logistic regression models after adjustment for study type, journal, country, and type of disease; estimates in bold are those statistically significant at 0.05 level.

^b^Reference category.

^c^Other: including in vivo and in vitro studies, case series, methodological analyses, qualitative studies, economic analyses, essays and viewpoints.

^d^Including 11 studies set in UK and other European countries.

^e^Mental health: including also brain diseases/abuse of substances; Digestive system and metabolic diseases: including also kidney, urinary diseases and sedentarism; Orthopedic diseases: including bones/soft tissue damages; Injuries and also dysplasia; Female reproductive system diseases: including also maternal, pregnancy and infant diseases.

### Time trend analysis

**Figure 1.**
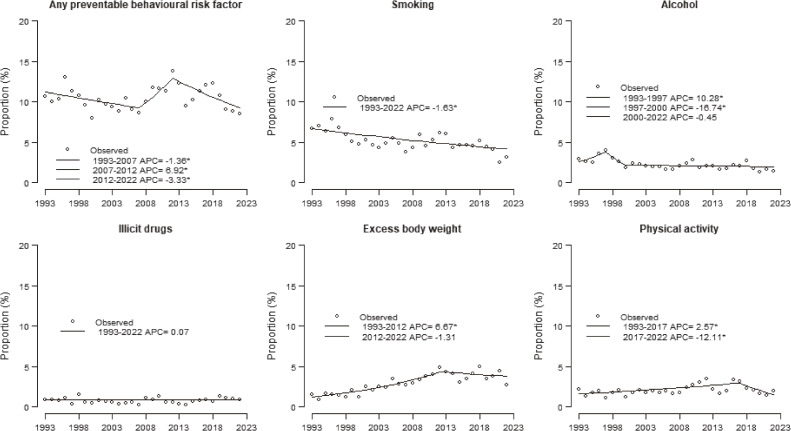
Joinpoint analyses^a^ for (*n* = 36,587) publications on preventable behavioral risk factors in JAMA, BMJ, the Lancet, and NEJM, all combined, in the years 1993–2022. ^a^Data were obtained from PubMed online database. Joinpoint regression models were fitted allowing for up to 6 joinpoints. ^*^Indicates that the annual percent change (APC) is significantly different from zero at the alpha = 0.05 level.

Figure 1 shows the joinpoint analyses of the proportion of articles focused on any preventable behavioral risk factor out of all the articles published in the four journals in the years 1993–2022. Considering JAMA, BMJ, the Lancet, and NEJM combined, we observed a statistically significant decrease in the proportion of papers dealing with preventable behavioral risk factors from 10.7% in 1993 to 8.6% in 2007 (APC −1.36%), a subsequent significant increase up to 13.8% in 2012 (APC +6.92%), followed by a significant decrease in the following years (APC −3.33%). The joinpoint analyses on publications on any preventable behavioral risk factor by each of the four selected journals (JAMA, BMJ, the Lancet, and NEJM) are reported in eFigure 1. Overall, in all the journals except BMJ, we observed a decrease in the proportion of papers dealing with preventable behavioral risk factors in the last years, with a more marked decline in the Lancet and NEJM.

**Figure 2.**
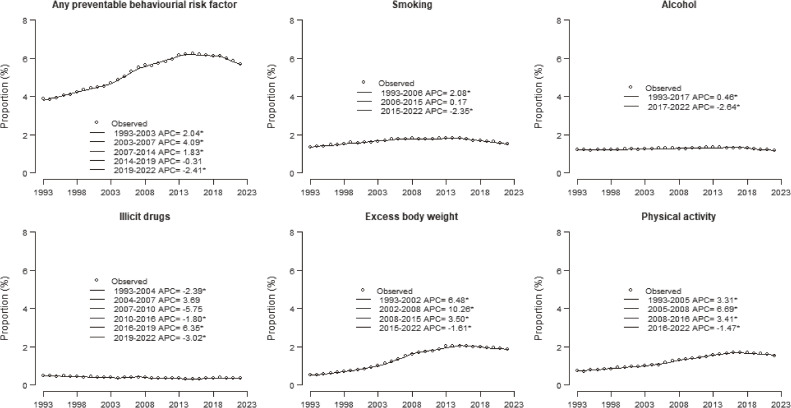
Joinpoint analyses^a^ for (*n* = 2,2983,246) publications on preventable behavioral risk factors in PubMed in the years 1993–2022. ^a^Data were obtained from PubMed online database. Joinpoint regression models were fitted allowing for up to 6 joinpoints. ^*^Indicates that the annual percent change (APC) is significantly different from zero at the alpha = 0.05 level.

Figure 2 shows the joinpoint analyses of the proportion of the articles focused on any preventable behavioral risk factor out of all the 22.98 million articles published in the whole medical literature between 1993 and 2022. The joinpoint analysis shows an initial increase in the proportion of papers dealing with any preventable behavioral risk factor between 3.9% in 1993 and 6.2% in 2014 (APC +2.04% in 1993–2003; APC +4.09% in 2003–2007; APC +1.83% in 2007–2014, all statistically significant), followed by a decrease (APC −0.31% not significant in 2014–2019; APC −2.41% statistically significant in 2019–2022).

eTable 6 shows the accuracy statistics to assess the validity of the search strings used by us to identify studies focused on various behavioral risk factors in the trend analyses, comparing the classification made applying search strings on publications from the four journals in 2022 with that obtained through the analysis of individual articles, considered as the “gold standard”. Overall, the search string designed to identify papers dealing with any preventable risk factor had a sensitivity of 0.856, a specificity of 0.982 and a Youden’s statistic of 0.837 (with Youden’s equal to 1 representing the optimal statistic performing the classification as the gold standard). For the search strings designed to identify the specific behavioral risk factors, the Youden’s statistic ranged between 0.577 and 0.863.

eTable 7, eTable 8, and eTable 9 show the numbers of publications on each preventable behavioral risk factor in the whole medical literature and in the four journals in 1993–2022, retrieved using the search strings in PubMed/MEDLINE.

## DISCUSSION

In 2022, 8% of publications from JAMA, BMJ, the Lancet, and NEJM dealt with smoking, alcohol drinking, use of illicit drugs, excess body weight, or physical activity. Extending the focus to the whole medical literature, in the last 30 years the proportion of publications dealing with preventable behavioral risk factors was even lower. Despite an initial increase up to 2014, we observe a decrease until the last year studied (2022). Studies on smoking, alcohol, excess body weight, and physical activity have all undergone to a reduction in proportion since 2015–2017, whereas those on illicit drugs started decreasing in popularity more recently (since 2019).

The main determinant of low interest of the scientific literature in behavioral risk factors is likely the lack of public funds allocated for preventive medicine. Indeed, in the European Union only 2.8% of total health expenditure in 2018 was devoted to preventive care.^17^ Moreover, a recent analysis revealed that of all the projects funded by United States NIH in 2019, only 0.8% dealt with tobacco, 0.6% with alcohol, 1.3% with substance abuse, 0.9% with obesity, and 0.4% with physical activity.^14^ Accordingly, these figures are even more critical if we consider research funds from the private sector, by far the main supporter of medical research. Indeed, private pharmaceutical corporates’ sponsorships often prioritize financial considerations, making it unlikely for these companies to support primary prevention studies that may not yield measurable profitability and cannot be patented. The strong interest of transnational corporations in the tobacco, alcohol, and food industries in promoting research on their products, thus on behavioral risk factors,^18^ combined with their inherent conflict of interest, often leads to results that may be biased.^19^^–^^21^ Thus, besides largely neglected, research on primary prevention is often impartial.^19^ Several studies have identified other barriers contributing to the low interest in prevention on a global scale. Among them, there is a limited request by the healthcare system: time constraints, heavy workload and a general skepticism on the effectiveness of preventive measures of healthcare providers all contribute to the scarce prioritization of preventive care.^8^^–^^11^^,^^22^^,^^23^ Furthermore, the inherent difficulties in quantifying the impact of preventive measures, coupled with the frequently suboptimal design of the studies aimed at such quantification,^13^ contribute to the relegation of prevention to a lower priority within the healthcare sector.

Our multivariable analysis revealed that studies from the United States more frequently address behavioral risk factors compared to those from other countries. A possible explanation is that this trend reflects the unique public health priorities in the United States, where obesity is a particularly significant public health issue compared to other countries.

Our trend analysis showed that in the past 30 years the proportion of articles on preventable behavioral risk factors increased until 2014 and decreased thereafter. The interest on lifestyles in the medical literature dates back to the late 19^th^ century^24^^,^^25^ and the first epidemiological studies quantifying the unfavorable impact of behavioral risk factors on specific health outcomes to 1950, when a direct association between tobacco smoking and lung cancer risk was first observed in analytical studies.^26^^,^^27^ The subsequent growing evidence of the mortality and morbidity burden attributable to major preventable risk factors resulted in an increased interest of the scientific community to investigate unhealthy lifestyles. This explains the increase in the volume of research devoted to modifiable risk factors in the whole medical literature observed before 2014. However, we observed a downward trend over the last decade. Except for BMJ, a decreasing trend was observed in all the main journals individually. For the Lancet, this can be totally or partially explained by the introduction of thematic journals specifically oriented to public health and prevention (eg, Lancet Global Health since 2013 and Lancet Public Health since 2016). Another reason that can partially explain the decreasing interest in primary prevention in the whole medical literature over the last years is the outbreak of the COVID-19 pandemic. Indeed, we observed that in 2022 the topic most considered by the four journals was COVID-19, and papers on COVID-19 less likely dealt with preventable behavioral risk factors. This, however, does not apply to the decreasing trend observed before the pandemic. Another possible reason for the downward trend in scientific articles on these specific preventable risk factors could be related to the shift towards new emerging risk factors, such as the use of electronic cigarettes, heated tobacco products, screen-based device and social media exposure, which should be explored in subsequent analyses.

### Limitations

A limitation of the study lies in the arbitrariness of the terms used in the search strings to analyze trends in publications on preventable behavioral risk factors. For example, the Youden’s index for the string related to excess body weight was relatively low, primarily due to its low sensitivity. This was likely due to the exclusion of certain keywords that could have identified a broader range of relevant articles, thereby affecting the specificity of the string. Nevertheless, the Youden’s statistics for the search strings demonstrate overall a relatively satisfactory performance, in particular for the string designed to identify any preventable behavioral risk factor. Another limitation is the arbitrary choice of considering only the publications with abstract available for both the analysis of the four journals and the trend analysis of the whole medical literature. However, given that articles without abstract represent less than 15% of all publications over the last decade, we do not expect substantially different results when considering the whole literature. Another limitation of the study concerns the appropriateness of considering as papers on prevention those studies evaluating interventions to decrease unhealthy habits (thus, behaviors considered as “outcome”). However, whether considering or not publications dealing with the behavior as an outcome, the proportion of papers on preventable risk factors remains low. An additional constraint of this study is our focus on a limited set of preventable risk factors. We deliberately chose these risk factors in order to focus attention on easily avoidable behavioral risk factors modifiable through an individual choice. For this reason, we are confident that they represent key targets for primary prevention efforts. For instance, we did not consider diet among the risk factors, since we found that constructing a search string for diet—while ensuring it specifically addressed diet as a preventable behavior—was particularly challenging and complex and because it was at least partially addressed by “obesity”. For this reason, we chose not to include it, which we acknowledge as an additional limitation of the study. Nonetheless, we have carefully selected risk factors that not only rank among the main causes of mortality morbidity and disabilities,^5^ but which also have consistently shown to be easily modifiable by individuals. Consequently, the active promotion of these behaviors holds the potential to yield prompt benefits to the overall population. Another limitation of the study is that we considered a decline in the volume of articles on preventable behavioral risk factors as a proxy for a decrease in interest in these topics. However, scientific interest was not directly assessed, so the interpretations of this study should be considered with caution.

For the first time, we evaluated the volume of medical literature devoted to specific preventable behavioral risk factors. Despite the central role of preventable behavioral risk factors for mortality, morbidity, and disability, research on these topics appears inadequate and the interest on these factors is mostly decreasing in time. The allocation of funds for primary prevention of avoidable risk factors, including tobacco smoking, alcohol drinking, use of illicit drugs, excess body weight, and physical inactivity, should be a priority to improve population health worldwide. Furthermore, primary prevention of lifestyle-related behaviors is becoming even more crucial due to the worldwide aging population, as these risk factors increasingly contribute to morbidity and disability in older adults.

## SUPPLEMENTARY MATERIAL

eTable 1. Search strings used (search date: March, 4^th^ 2024) to identify papers on selected preventable behavioral risk factors (ie, smoking, alcohol drinking, use of illicit drugs, excess body weight, and physical activity) published in PubMed between 1993–2022

eTable 2. Details on the 1,128 publications from JAMA, BMJ, Lancet, and NEJM, published in 2022

eTable 3. Characteristics of the 1,128 included articles published in JAMA, BMJ, Lancet, and NEJM in 2022

eTable 4. Characteristics of the 1,128 included articles published in JAMA, BMJ, Lancet, and NEJM in 2022, according to their focus on any preventable behavioural risk factors, smoking, alcohol use, use of illicit drugs, excess body weight, or physical activity

eTable 5. Distribution of papers focusing on selected preventable behavioral risk factors (ie, smoking, alcohol drinking, use of illicit drugs, excess body weight and physical activity) and percentages on the total of 1,128 articles published in JAMA, BMJ, Lancet, and NEJM in 2022, according to the role of the risk factor (outcome, exposure, burden or recommendation) in the paper

eTable 6. Accuracy statistics (sensitivity, specificity and Youden’s J statistics^a^) referring to the comparison of the classification of papers on selected preventable behavioral risk factors obtained applying search strings in PubMed/MEDLINE versus that obtained manually, considered as the “gold standard”, on publications from JAMA, BMJ, the Lancet, and NEJM in 2022

eTable 7. Number of publications (with abstract available) on each preventable behavioral risk factor from all medical literature retrieved using search strings in PubMed/MEDLINE in the years 1993–2022

eTable 8. Number of publications (with abstract available) on each preventable behavioral risk factor from Lancet and JAMA retrieved using search strings in PubMed/MEDLINE in the years 1993–2022

eTable 9. Number of publications (with abstract available) on each preventable behavioral risk factor from the BMJ and the New England Journal of Medicine retrieved using search strings in PubMed/MEDLINE in the years 1993–2022

eFigure 1. Joinpoint analyses^a^ for proportion of publications on preventable behavioural risk factors in JAMA, BMJ, the Lancet, and the NEJM, in the years 1993–2022
