# Supplementary material for: Do Not Let the Guard Down on Preventable Behavioral Risk Factors
Source: J Epidemiol. 2025 Nov 5;35(11):465–71. doi: 10.2188/jea.JE20250037 (PMC12527404; doi:10.2188/jea.JE20250037)
Supplement: Supplementary file 1 [file je-35-465-s001.pdf]

eTable 1. Search strings used (search date: March, 4th 2024) to identify papers on selected preventable behavioral risk factors (ie, smoking, alcohol drinking, use of illicit drugs, excess body weight, and physical activity) published in PubMed between 1993–2022

**Preventable behavioral risk factors**	**Search String**
Any preventable behavioral risk factor	tobacco[tiab] or smok*[tiab] or smok*[MeSH] or tobacco[mesh] OR alcohol[tiab] OR "alcoholic beverages"[MeSH Terms] OR "alcohol drinking"[MeSH Terms] OR (((((((((((((((cannabis[MeSH Terms]) OR (Hallucinogens[MeSH Terms])) OR (Opioid-Related Disorders[MeSH Terms])) OR (Substance Abuse Treatment Centers[MeSH Terms])) OR (Marijuana Abuse[MeSH Terms])) OR (Opiate Overdose[MeSH Terms])) OR (Marijuana Use[MeSH Terms])) OR ("Amphetamine-Related Disorders "[MeSH Terms])) OR (Narcotic-Related Disorders[MeSH Terms])) OR (Neonatal Abstinence Syndrome[MeSH Terms])) OR (Psychoses, Substance-Induced[MeSH Terms])) OR (Inhalant Abuse[MeSH Terms])) OR (Substance Abuse, Intravenous[MeSH Terms])) OR (Substance Abuse, Oral[MeSH Terms])) OR (Cocaine*[MeSH Terms])) OR (Heroin[MeSH Terms]))) OR (overweight[MeSH Terms] or obesity[tiab]) OR (Exercise[MeSH Terms] or physical activity[tiab])
Smoking	tobacco[tiab] or smok*[tiab] or smok*[MeSH] or tobacco[mesh]
Alcohol drinking	alcohol[tiab] OR "alcoholic beverages"[MeSH Terms] OR "alcohol drinking"[MeSH Terms]
Illicit Drugs	(((((((((((((((cannabis[MeSH Terms]) OR (Hallucinogens[MeSH Terms])) OR (Opioid-Related Disorders[MeSH Terms])) OR (Substance Abuse Treatment Centers[MeSH Terms])) OR (Marijuana Abuse[MeSH Terms])) OR (Opiate Overdose[MeSH Terms])) OR (Marijuana Use[MeSH Terms])) OR ("Amphetamine-Related Disorders "[MeSH Terms])) OR (Narcotic-Related Disorders[MeSH Terms])) OR (Neonatal Abstinence Syndrome[MeSH Terms])) OR (Psychoses, Substance-Induced[MeSH Terms])) OR (Inhalant Abuse[MeSH Terms])) OR (Substance Abuse, Intravenous[MeSH Terms])) OR (Substance Abuse, Oral[MeSH Terms])) OR (Cocaine*[MeSH Terms])) OR (Heroin[MeSH Terms]))
Excess body weight	overweight[MeSH Terms] or obesity[tiab]
Physical activity	exercise[MeSH Terms] or physical activity[tiab]

MeSH, Medical Subject Headings; tiab, title and abstract.

eTable 2. Details on the 1,128 publications from JAMA, BMJ, Lancet, and NEJM, published in 2022

Authors	Journal	Risk factor(s) [role]	Study Type	Study Design[a]	Geographic zone	PMID
Rubino et al.	JAMA	Excess body weight; Physical activity [Outcome]	Epi I	RCT	North America	35015037
Adrogué et al.	JAMA		Review	Review	NA	35852524
Cowan et al.	JAMA		Review	Review	North America	35103762
Freund et al.	JAMA		Review	Review	North America	36194215
Bhatt et al.	JAMA		Review	Review	NA	35166796
Ruopp, Cockrill	JAMA		Review	Review	North America	35412560
Walker, Shane	JAMA		Review	Review	NA	36282253
Carey et al.	JAMA	Alcohol; Excess body weight; Physical activity [Exposure]	Review	Review	North America	36346411
Gallaher, Charles	JAMA		Review	Review	North America	35258527
Kanner, Bicchi	JAMA		Review	Review	NA	35380580
Katz et al.	JAMA		Review	Review	North America	35503342
Kavanagh et al.	JAMA		Review	Review	North America	35788790
Lv et al.	JAMA		Epi I	RCT	Multicontinent	35579642
Mazzone, Lam	JAMA		Review	Review	North America	35040882
Li et al.	JAMA		Epi I	RCT	Asia	34928310
Hasbun	JAMA		Review	Review	NA	36472590
Tuddenham et al.	JAMA		Review	Review	North America	35015033
Sekeres, Taylor	JAMA		Review	Review	North America	36066514
Szuhany, Simon	JAMA		Review	Review	North America	36573969
Rigotti et al.	JAMA	Smoking [Outcome]	Review	Review	North America & UK	35133411
Räber et al.	JAMA		Epi I	RCT	Europe (UK excluded)	35368058
Sidhu et al.	JAMA		Epi I	RCT	Oceania	35997730
Gao et al.	JAMA		Epi I	RCT	Asia	35943472
Daily et al.	JAMA		Review	Review	North America	35916842
Belperio et al.	JAMA		Review	Review	North America	35230389
Dahl et al.	JAMA		Epi I	RCT	Multicontinent	35133415
Cheng et al.	JAMA		Epi I	RCT	Multicontinent	36166026
Mangione et al.	JAMA		Review	Review	North America	35997723
Russell et al.	JAMA		Epi I	RCT	North America	35707974
Balboni et al.	JAMA		Review	Review	North America	35819420
Aminian et al.	JAMA	Excess body weight [Exposure]	Epi O	Cohort	North America	35657620
Renú et al.	JAMA		Epi I	Phase 1/2 study	Europe (UK excluded)	35143603
Currie et al.	JAMA		Review	Review	North America	35762992
Sharma	JAMA	Smoking [Exposure]	Review	Review	North America	35972481
Mangione et al.	JAMA		Review	Review	North America	35727271
Wulf Hanson et al.	JAMA		Review	Review	NA	36215063
Marcus	JAMA		Other	Other	North America	36413229
Lenze et al.	JAMA	Physical activity [Exposure]	Epi I	RCT	North America	36511926
Davidson et al.	JAMA		Review	Review	North America	35471505
Bagg et al.	JAMA		Epi I	RCT	Oceania	35916848
Catto et al.	JAMA		Epi I	RCT	UK	35569079
Qiu et al.	JAMA		Epi I	RCT	Asia	35943471
Kaye et al.	JAMA		Epi I	RCT	Multicontinent	36194218
Bastarache et al.	JAMA		Review	Review	North America	34982132
Oslin et al.	JAMA		Epi I	RCT	North America	35819423
Preskorn et al.	JAMA		Epi I	RCT	North America	35191924
Belohlavek et al.	JAMA		Epi I	RCT	Europe (UK excluded)	35191923
Tang et al.	JAMA		Epi I	RCT	Asia	35997729
Perkins et al.	JAMA		Epi I	RCT	UK	35072713
Walter, Malani	JAMA		Other	Other	North America	36374512
Butcher et al.	JAMA		Review	Review	NA	36511921
Chang et al.	JAMA		Epi O	Cohort	North America	35608581
Cipolletta et al.	JAMA		Epi O	Cohort	UK	35916846
Bell et al.	JAMA		Epi O	Cohort	North America	35040888
Stahl et al.	JAMA		Epi I	RCT	Multicontinent	35881122
Naggie et al.	JAMA		Epi I	RCT	North America	36269852
Ramnarayan et al.	JAMA		Epi I	RCT	UK	35390113
Mehra et al.	JAMA		Epi O	Cohort	North America	36074476
Marrouche et al.	JAMA		Epi I	RCT	North America	35727277
Alhazzani et al.	JAMA		Epi I	RCT	Multicontinent	35569448
Baman, Eskandari	JAMA		Other	Other	NA	36511924
Walter, Malani	JAMA		Other	Other	NA	36112391

Authors	Journal	Risk factor(s) [role]	Study Type	Study Design[a]	Geographic zone	PMID
Basch et al.	JAMA		Epi I	RCT	North America	35661856
Shi et al.	JAMA		Epi I	RCT	Asia	35881121
Bösel et al.	JAMA		Epi I	RCT	Europe (UK excluded)	35506515
Toff et al.	JAMA		Epi I	RCT	UK	35579641
Hernán et al.	JAMA		Other	Methodological study	NA	36508210
Devauchelle-Pensec et al.	JAMA		Epi I	RCT	Europe (UK excluded)	36125471
Accorsi et al.	JAMA		Epi O	Case-control study	North America	35060999
Chappell et al.	JAMA		Epi I	RCT	UK	35503345
Ramnarayan et al.	JAMA		Epi I	RCT	UK	35707984
O'Connor et al.	JAMA		Review	Systematic review	NA	35727272
Myburgh et al.	JAMA		Epi I	RCT	Oceania	36286097
Oster et al.	JAMA		Epi O	Cohort	North America	35076665
Levy et al.	JAMA		Epi I	RCT	Europe (UK excluded)	35103766
Hara et al.	JAMA		Epi I	RCT	Europe (UK excluded)	36255427
Guglieri et al.	JAMA		Epi I	RCT	Multicontinent	35381069
Bradbury et al.	JAMA		Epi I	RCT	Multicontinent	35315874
Meersch et al.	JAMA		Epi I	RCT	Europe (UK excluded)	35665794
Gupta et al.	JAMA		Epi I	RCT	Multicontinent	35285853
Metz et al.	JAMA		Epi O	Cohort	North America	35129581
Goodwin et al.	JAMA		Epi I	RCT	Multicontinent	35608580
Messier et al.	JAMA	Excess body weight [Exposure]	Epi I	RCT	North America	36511925
Magnus et al.	JAMA		Epi O	Cohort	Europe (UK excluded)	35323851
Chen et al.	JAMA		Epi I	RCT	Asia	35972485
Shlipak et al.	JAMA		Other	Case series	North America	35939309
Ekström et al.	JAMA		Epi I	RCT	Oceania	36413230
Tucker et al.	JAMA		Epi I	RCT	UK	35503346
Kim et al.	JAMA		Epi O	Cohort	Asia	35867050
Nissen et al.	JAMA		Epi I	Phase 1/2 study	Multicontinent	35368052
Maslo et al.	JAMA		Other	Case series	Africa	34967859
Frat et al.	JAMA		Epi I	RCT	Europe (UK excluded)	36166027
Cabrera et al.	JAMA		Other	Other	NA	36346422
Pesonen et al.	JAMA		Epi I	RCT	Europe (UK excluded)	35852528
Fielding-Singh et al.	JAMA		Epi O	Cohort	North America	36326747
Jabagi et al.	JAMA		Epi O	Cohort	Europe (UK excluded)	34807248
Chang et al.	JAMA		Epi O	Cohort	North America	36573973
Simon et al.	JAMA		Epi I	RCT	North America	35166800
Hammond et al.	JAMA		Review	Systematic review	NA	36286098
Aggarwal et al.	JAMA	Excess body weight [Burden]	Epi O	Cross sectional study	North America	35997731
Lewis et al.	JAMA	Physical activity [Outcome]	Epi I	RCT	Multicontinent	35852527
Fell et al.	JAMA		Epi O	Cohort	North America	35323842
Desai et al.	JAMA		Other	Case series	North America	35994281
Li et al.	JAMA		Epi O	DB analysis	North America	35103775
Schlapbach et al.	JAMA		Epi I	RCT	Multicontinent	35759691
Demirjian et al.	JAMA		Epi O	Cohort	North America	35258532
McClymont et al.	JAMA		Epi O	Cohort	North America	35499852
Sandner et al.	JAMA		Review	Systematic review	NA	35943473
Lebwohl et al.	JAMA		Epi I	RCT	North America	36125472
Wang et al.	JAMA		Epi O	Cohort	North America	35315884
Goldenberg et al.	JAMA		Epi I	RCT	North America	35015038
Schottinger et al.	JAMA		Epi O	Cohort	North America	35670788

Authors	Journal	Risk factor(s) [role]	Study Type	Study Design[a]	Geographic zone	PMID
Azzolini et al.	JAMA		Epi O	Cohort	Europe (UK excluded)	35796131
Mangione et al.	JAMA		Review	Systematic review	NA	36219440
Nelson et al.	JAMA		Review	Systematic review	NA	36318133
Doyle et al.	JAMA		Epi I	RCT	North America	36472596
Lo Re et al.	JAMA		Epi O	Cohort	North America	35972486
Bernard et al.	JAMA		Epi I	RCT	Oceania	36286192
Fleming-Dutra et al.	JAMA		Epi O	Case-control study	North America	35560036
Heitzman	JAMA		Other	Other	NA	36472591
Kurihara et al.	JAMA		Other	Case series	North America	35085383
Pérez de la Ossa et al.	JAMA		Epi I	RCT	Europe (UK excluded)	35510397
Heesakkers et al.	JAMA		Epi O	Cohort	Europe (UK excluded)	35072716
Calvert	JAMA		Other	Other	NA	36255424
Venkatesh et al.	JAMA		Epi O	Cross sectional study	North America	35412565
Faust et al.	JAMA		Other	Methodolo gical study	North America	35594035
Sutton et al.	JAMA		Epi I	RCT	North America	35258533
Huggins et al.	JAMA		Epi O	Cross sectional study	North America	35103767
Kam et al.	JAMA		Epi O	Cohort	North America	35143601
Eldridge et al.	JAMA		Epi O	Cross sectional study	North America	35819424
Jorge et al.	JAMA		Epi O	Cohort	North America	36112387
Friedman et al.	JAMA	Illicit drugs [Burden]	Epi O	DB analysis	North America	35412573
He et al.	JAMA		Epi O	Cross sectional study	North America	35819433
O'Brien et al.	JAMA		Epi I	RCT	Multicontinent	35029629
Cohen et al.	JAMA		Epi I	RCT	North America	36260754
Guirguis-Blake et al.	JAMA		Review	Systematic review	NA	35471507
Mangione et al.	JAMA	Excess body weight [Burden]	Review	Review	North America	36098719
van der Vaart et al.	JAMA		Epi I	RCT	Europe (UK excluded)	36538310
Donohue et al.	JAMA		Review	Review	North America	36125468
Mangione et al.	JAMA	Smoking; Excess body weight; Physical activity [Recommendation]	Review	Review	North America	35881115
Chou et al.	JAMA		Review	Systematic review	NA	35997724
Pirruccello et al.	JAMA		Epi O	Cohort	UK	36378208
Bearne et al.	JAMA	Physical activity [Recommendation]	Epi I	RCT	North America	35412564
Johnston et al.	JAMA		Epi O	Cohort	North America	36272098
Adhikari et al.	JAMA		Epi O	Cohort	North America	35325015
Gazendam et al.	JAMA		Epi I	RCT	North America	36194219
Jonas et al.	JAMA	Excess body weight [Burden]	Review	Systematic review	NA	36098720
Mangione et al.	JAMA		Review	Systematic review	NA	36378202
McConnell et al.	JAMA		Epi I	RCT	North America	35788794
White et al.	JAMA	Alcohol [Burden]	Epi O	DB analysis	North America	35302593
Johnson et al.	JAMA		Review	Systematic review	NA	35699701
James et al.	JAMA		Epi I	RCT	North America	36066520
Choudhry et al.	JAMA		Epi I	RCT	North America	36538309
Marcus, Walter	JAMA		Other	Other	NA	36472593
Berger et al.	JAMA		Epi I	RCT	Multicontinent	35040887
Mangione et al.	JAMA		Review	Systematic review	NA	36318127

Authors	Journal	Risk factor(s) [role]	Study Type	Study Design[a]	Geographic zone	PMID
Kelly	JAMA		Other	Other	NA	36573970
Brekke-Riedl	JAMA		Other	Other	NA	36413227
Florig et al.	JAMA		Epi O	DB analysis	North America	36048452
Cohen, Teal	JAMA		Other	Other	NA	35984667
Feltner et al.	JAMA		Review	Systematic review	NA	36378203
Castro-Frenzel	JAMA		Other	Other	NA	36538307
Gutiérrez et al.	JAMA		Epi O	Cohort	North America	35667006
Ray et al.	JAMA	Excess body weight [Exposure]	Epi O	Cross sectional study	North America	35076678
Wellbery	JAMA		Other	Other	NA	36318130
Florian et al.	JAMA		Epi I	RCT	North America	36219407
Belkin et al.	JAMA		Other	Other	NA	36107415
Gartlehner et al.	JAMA		Review	Systematic review	North America	36318128
Jin	JAMA		Other	Other	North America	36166024
McDermott et al.	JAMA	Physical activity [Outcome]	Epi I	RCT	North America	36194220
Arabi et al.	JAMA		Epi I	RCT	Asia	36125473
Kelly et al.	JAMA		Epi O	Cohort	North America	36156706
Walter	JAMA		Other	Other	NA	36346414
Green	JAMA		Review	Review	NA	36573994
Benvenuto	JAMA		Other	Other	NA	36511922
Karches	JAMA		Other	Other	NA	36346412
Daum	JAMA		Other	Other	NA	36378205
Fernando et al.	JAMA		Epi O	Cohort	North America	36286084
Walter	JAMA		Other	Other	North America	36166025
Suran	JAMA		Other	Other	NA	36573980
Stark et al.	JAMA		Other	Other	NA	35960529
Butcher et al.	JAMA		Review	Review	NA	36512367
Kutscher	JAMA		Other	Other	NA	36094525
Davidson et al.	JAMA		Review	Systematic review	NA	35076659
Forrest et al.	JAMA		Epi O	Cohort	North America & UK	35076666
Grossman, Verma	JAMA		Other	Other	North America	36318121
Marcus	JAMA		Other	Other	NA	36573972
Jin	JAMA		Other	Other	North America	36378207
Binger et al.	JAMA		Other	Other	North America	36251309
Im	JAMA		Other	Other	NA	36282254
Kornblith et al.	JAMA		Epi O	Cohort	North America	35438728
Soriano et al.	JAMA		Epi O	Cross sectional study	Oceania	35788795
Siddharthan et al.	JAMA	Smoking [Exposure]	Epi O	Cross sectional study	Multicontinent	35015039
Watson et al.	JAMA		Epi O	Cohort	North America	35230393
Anderson et al.	JAMA		Other	Other	North America	36190725
Mangione et al.	JAMA		Review	Systematic review	NA	36219403
Koh, Fiore	JAMA	Smoking [Recommendation]	Other	Other	NA	36331445
Dusetzina et al.	JAMA		Epi O	Cross sectional study	North America	36255428
Ahmed et al.	JAMA		Epi O	Cross sectional study	North America	36573974
Jin	JAMA		Other	Other	North America	35997728
Spitzer et al.	JAMA		Epi O	Cohort	Asia	35006256
Suran	JAMA		Other	Other	NA	36044242
Halpern et al.	JAMA		Review	Review	North America	36413249
Fleming	JAMA		Other	Other	NA	36194216
Rosen, Beyrer	JAMA		Other	Other	North America	36318218
Jin	JAMA		Other	Other	North America	36219419
Dorabawila et al.	JAMA		Epi O	DB analysis	North America	35559959
Mafi et al.	JAMA		Epi O	Cohort	North America	35040886
Davidson et al.	JAMA		Review	Systematic review	NA	35289876
Auger et al.	JAMA		Other	Other	North America	36201185
Shah et al.	JAMA		Epi O	Cohort	Oceania	35315886

Authors	Journal	Risk factor(s) [role]	Study Type	Study Design[a]	Geographic zone	PMID
Bruxvoort et al.	JAMA		Epi O	Cohort	North America	35333303
Mangione et al.	JAMA		Review	Systematic review	NA	35608574
Archer	JAMA		Other	Other	NA	36166022
Khullar	JAMA		Other	Other	NA	36149664
Sakran et al.	JAMA		Other	Other	North America	36166018
Shahmoradi et al.	JAMA		Epi O	DB analysis	North America	36409512
Parcha et al.	JAMA		Epi O	Cohort	North America	35377943
Cullen et al.	JAMA		Other	Other	NA	36053542
Parks et al.	JAMA		Epi O	DB analysis	North America	35258534
Song	JAMA		Other	Other	North America	36166013
Pawar et al.	JAMA		Epi O	Cohort	North America	35289881
Crear-Perry et al.	JAMA		Other	Other	North America	36318120
Rome et al.	JAMA		Epi O	DB analysis	North America	35670795
Fernandez Lynch, Sachs	JAMA		Other	Other	North America	36480185
Perl et al.	JAMA		Other	Case series	North America	36355384
Jin	JAMA		Other	Other	North America	36318132
Iio, Ishida	JAMA		Other	Case series	Asia	36441545
Meeks et al.	JAMA		Epi O	Cross sectional study	North America	35951317
Webber et al.	JAMA		Review	Systematic review	NA	35536261
Romero, Ramón Michel	JAMA		Other	Other	Other American countries	36318122
Behr et al.	JAMA		Epi O	Cohort	North America	35119452
Udalova et al.	JAMA		Epi O	Cross sectional study	North America	36573975
Thompson et al.	JAMA		Epi O	Cohort	North America	36255426
Lamprea-Montealegre et al.	JAMA		Epi O	Cross sectional study	North America	36066519
Park et al.	JAMA		Other	Methodological study	NA	34982138
Mitchell, Aronson	JAMA		Other	Other	North America	36166009
Cook, Donohue	JAMA		Other	Other	North America	36166010
Holmberg, Andersen	JAMA		Other	Other	NA	36394881
Mangione et al.	JAMA		Review	Systematic review	NA	35608838
Reingold et al.	JAMA		Other	Other	North America	36318123
Lin et al.	JAMA		Epi O	Cohort	North America	36155617
Simon et al.	JAMA		Other	Other	NA	36136366
Grunau et al.	JAMA		Epi O	Cohort	North America	34860253
Chou et al.	JAMA		Review	Systematic review	NA	35608575
Jollis et al.	JAMA		Epi O	Cross sectional study	North America	36335474
Plappert et al.	JAMA	Smoking [Exposure]	Other	Case series	Europe (UK excluded)	36315193
Kerlikowske et al.	JAMA		Epi O	Cohort	North America	35699706
Azoulay et al.	JAMA		Epi O	Cohort	Europe (UK excluded)	35179564
Suran	JAMA		Other	Other	North America	36083593
Kuehn	JAMA		Other	Other	North America	36301578
Davidson et al.	JAMA		Other	Other	North America	35315879
Chua et al.	JAMA		Other	Economic study	North America	35024763
Galea, Abdalla	JAMA		Other	Other	North America	36166016
Hargreave et al.	JAMA		Epi O	Cohort	Europe (UK excluded)	34982120
Etminan et al.	JAMA		Epi O	DB analysis	North America	36511932
McPherson	JAMA		Other	Other	North America	36166017
Morral, Smart	JAMA		Other	Other	North America	36166014
Gans et al.	JAMA		Epi O	Cohort	North America	34994775
Peachman	JAMA		Other	Other	North America	36318134

Authors	Journal	Risk factor(s) [role]	Study Type	Study Design[a]	Geographic zone	PMID
Alejo et al.	JAMA		Epi O	Cross sectional study	North America	35113143
Suran	JAMA		Other	Other	North America	36260342
Varma, Goldenberg	JAMA		Other	Other	North America	36342708
Hwang et al.	JAMA		Other	Other	NA	36279114
Edmonson et al.	JAMA		Epi O	Cohort	North America	35727278
Viswanathan et al.	JAMA		Review	Systematic review	NA	36219404
Liu, Wadhera	JAMA		Epi O	DB analysis	North America	36378215
Li et al.	JAMA		Epi O	Cohort	UK	35943470
Dehmer et al.	JAMA		Other	Methodological study	North America	35471506
Robinson	JAMA		Other	Other	North America	36355357
Amaral et al.	JAMA		Epi O	Cohort	North America	35916847
Dar et al.	JAMA		Other	Other	North America	36374488
Duffy et al.	JAMA		Other	Other	North America	35727286
Mangione et al.	JAMA		Review	Systematic review	NA	35536260
Gomes et al.	JAMA	Illicit drugs [Outcome]	Epi O	Cohort	North America	35230394
Johnston et al.	JAMA		Other	Other	NA	36206010
MacDonald et al.	JAMA		Other	Other	North America	36318126
Suran	JAMA		Other	Other	North America	36103202
White et al.	JAMA		Epi O	DB analysis	North America	36318197
Britton et al.	JAMA		Epi O	Case-control study	North America	35157002
Shook et al.	JAMA		Other	Other	NA	35129576
Ke et al.	JAMA		Epi O	DB analysis	North America	36239969
Fernandes et al.	JAMA		Other	Other	North America	34854872
Picone et al.	JAMA		Epi O	DB analysis	Multicontinent	35166811
Alexander, Mansour	JAMA	Illicit drugs [Burden]	Other	Other	North America	36306147
Rubin	JAMA		Other	Other	North America	36166033
Haddad et al.	JAMA		Other	Other	NA	36454555
Howard et al.	JAMA		Epi O	DB analysis	North America	35230401
Genzen et al.	JAMA		Epi O	Cross sectional study	North America	36413243
Rader et al.	JAMA		Epi O	Cross sectional study	North America	36318194
Hahn et al.	JAMA		Epi I	RCT	North America	34982119
Bates et al.	JAMA		Epi O	Case-control study	North America	34914825
Patnode et al.	JAMA	Excess body weight; Physical activity [Recommendation]	Review	Systematic review	NA	35881116
O'Connor et al.	JAMA		Epi I	RCT	Oceania	36066518
Richman, Schulman	JAMA		Other	Other	NA	36394908
Rubin	JAMA		Other	Other	NA	36129724
Borrero et al.	JAMA		Other	Other	NA	36318124
Ulloa et al.	JAMA		Epi O	Cohort	North America	35175280
Jin	JAMA		Other	Other	North America	36219439
Pronovost et al.	JAMA		Other	Other	NA	35212725
Suran	JAMA		Other	Other	North America	36173620
Williams et al.	JAMA		Epi O	DB analysis	North America	35699714
Abbasi	JAMA		Other	Other	North America	36416851
Cruz Rivera et al.	JAMA		Review	Systematic review	NA	35579638
Tucci, Califf	JAMA		Other	Other	North America	36459164
Feltner et al.	JAMA		Review	Systematic review	NA	35289875
Spector-Bagdady et al.	JAMA		Other	Other	North America	35916854
Frieden, McClelland	JAMA		Other	Other	NA	36206014
Zhang, Anderson	JAMA		Other	DB analysis	North America	36315190

Authors	Journal	Risk factor(s) [role]	Study Type	Study Design[a]	Geographic zone	PMID
Nelson et al.	JAMA		Other	Other	North America	35972493
Miech et al.	JAMA	Smoking [Outcome]	Epi O	DB analysis	North America	35315899
Cohen et al.	JAMA		Epi O	DB analysis	North America	35881132
Chou et al.	JAMA		Review	Systematic review	NA	35608842
Cunningham et al.	JAMA		Other	Other	North America	36166011
Matta, Nicholas	JAMA		Epi O	DB analysis	North America	36121675
Mariño-Ramírez et al.	JAMA		Epi O	DB analysis	North America	36538317
Suran	JAMA		Other	Other	NA	36350667
Kader, Chebli	JAMA		Other	Other	NA	36136351
Kahwati et al.	JAMA		Review	Systematic review	NA	35076660
Hudson et al.	JAMA		Review	Systematic review	NA	36098725
Bowe et al.	JAMA		Epi O	DB analysis	North America	35763005
Friedensohn et al.	JAMA		Epi O	Cohort	Asia	35297962
Walker et al.	JAMA		Other	Other	NA	36318125
Cox et al.	JAMA	Smoking [Outcome]	Epi I	RCT	North America	35699705
Rubin	JAMA		Other	Other	North America	36223128
Tan et al.	JAMA		Epi O	Cohort	Asia	35147657
Davenport et al.	JAMA		Other	Methodological study	NA	35679081
De Boer et al.	JAMA		Epi O	DB analysis	North America	35997744
Liu et al.	JAMA	Illicit drugs [Outcome]	Epi O	DB analysis	North America	35536272
Cram et al.	JAMA		Other	Other	NA	36201190
Waggoner et al.	JAMA		Epi O	Cross sectional study	North America	36018570
Harlow et al.	JAMA	Illicit drugs [Burden]	Other	Other	North America	36331491
Lamkin	JAMA		Other	Other	North America	36190715
Han et al.	JAMA	Smoking [Burden]	Epi O	Cross sectional study	North America	35471512
Abbasi	JAMA		Other	Other	NA	36368010
Hubert et al.	JAMA		Epi I	Controlled trial	Europe (UK excluded)	35510389
Bruzelius, Martins	JAMA	Illicit drugs [Burden]	Epi O	DB analysis	North America	36472602
Eisenberg et al.	JAMA		Epi O	DB analysis	North America	36121674
Aaron et al.	JAMA		Other	Other	North America	36480209
Johnson, Berwick	JAMA		Other	Other	North America	36409483
Shashikumar et al.	JAMA		Epi O	Cross sectional study	North America	36282256
Dickerson Mayes et al.	JAMA		Epi O	DB analysis	North America	36053549
Hipp et al.	JAMA		Epi O	DB analysis	North America	35380591
Barry, Tseng	JAMA		Other	Other	North America	36178699
Nieman et al.	JAMA		Epi I	RCT	North America	36538311
Kaufman, Delgado	JAMA		Other	Other	North America	36166012
Webber et al.	JAMA		Epi O	DB analysis	North America	36094572
Mengesha et al.	JAMA		Other	Other	North America	36318119
Gardner	JAMA		Other	Other	North America	36170054
Jørgensen et al.	JAMA		Epi O	DB analysis	Europe (UK excluded)	35254379
Hazra et al.	JAMA		Epi O	DB analysis	North America	36178700
Kuehn	JAMA	Illicit drugs [Burden]	Other	Other	NA	36069924
Goralnick et al.	JAMA		Other	Other	Europe (UK excluded)	36469331
Hoerster et al.	JAMA	Excess body weight; Physical activity [Outcome]	Epi I	RCT	North America	36511927
Edwards et al.	JAMA		Epi O	Cohort	Multicontinent	35315885
Hynes et al.	JAMA		Other	Other	NA	36170060

Authors	Journal	Risk factor(s) [role]	Study Type	Study Design[a]	Geographic zone	PMID
Haque et al.	JAMA		Other	Economic study	North America	35670796
Mangione et al.	JAMA		Review	Review	NA	36166020
Nori-Sarma et al.	JAMA		Epi O	DB analysis	North America	36538316
Cohen et al.	JAMA		Other	Other	North America	36279127
Choi et al.	JAMA		Epi O	DB analysis	North America	35881133
Levy et al.	JAMA		Epi O	Cohort	Asia	35588048
Tsay et al.	JAMA		Epi O	DB analysis	North America	35394497
Baig, Dusetzina	JAMA		Epi O	DB analysis	North America	36368009
Viswanathan et al.	JAMA		Review	Systematic review	NA	36219399
Barnett et al.	JAMA		Epi O	DB analysis	North America	36036916
Bhavani et al.	JAMA		Epi O	Cross sectional study	North America	36066526
Levy et al.	JAMA		Epi O	Cohort	Europe (UK excluded)	34928295
Okike et al.	JAMA		Epi O	DB analysis	North America	34967845
Ruder	JAMA		Other	Other	North America	36166032
Tian et al.	JAMA		Other	Case series	Asia	36399333
Mangione et al.	JAMA		Other	Other	North America	36251304
Venkatesh et al.	JAMA		Epi O	Cross sectional study	North America	35972487
Gonzales, Deal	JAMA		Epi O	DB analysis	North America	35438737
Jones et al.	JAMA		Epi O	Cross sectional study	North America	35696249
Suran	JAMA		Other	Other	NA	35976681
Birnie et al.	JAMA		Epi O	Cohort	Europe (UK excluded)	35913747
Madhusoodanan	JAMA		Other	Other	NA	36322069
Bradley et al.	JAMA		Epi O	DB analysis	North America	35394487
Rubin	JAMA		Other	Other	NA	36449325
Rubin	JAMA		Other	Other	NA	36383395
Razzaghi et al.	JAMA		Epi O	DB analysis	North America	35452085
Schwandt et al.	JAMA		Epi O	DB analysis	North America	35797033
Larkin	JAMA		Other	Other	NA	36383462
Wang et al.	JAMA		Epi O	Cohort	North America	35050314
Fang et al.	JAMA	Excess body weight [Exposure]	Epi O	Cross sectional study	North America	35579650
Ridgway et al.	JAMA		Epi O	Case-control study	North America	36149677
Cambonie et al.	JAMA		Epi O	Cohort	Europe (UK excluded)	35788802
Wunnava et al.	JAMA		Epi O	DB analysis	North America	35943478
Rewers et al.	JAMA		Epi O	Cohort	Multicontinent	35930271
Sarzynski et al.	JAMA		Epi O	Cohort	North America	35089309
Lo	JAMA		Other	Other	NA	36170057
Cardoso et al.	JAMA		Epi O	Cross sectional study	Other American countries	35749125
Klompas et al.	JAMA		Epi O	DB analysis	North America	35704347
Webster, Gostin	JAMA		Other	Other	North America	36166019
Dai et al.	JAMA	Smoking [Exposure]	Epi O	DB analysis	North America	36346420
Abbasi	JAMA		Other	Other	North America	36197671
Akashi et al.	JAMA		Epi O	Cohort	Asia	35113161
Nattino et al.	JAMA		Chemical/toxicologic	Chemical study	Europe (UK excluded)	35363259

Authors	Journal	Risk factor(s) [role]	Study Type	Study Design[a]	Geographic zone	PMID
			al, in vivo, in vitro			
Landon et al.	JAMA		Epi O	Cohort	North America	36472594
Schaubel et al.	JAMA		Epi O	Cohort	North America	35994263
Bond et al.	JAMA		Epi O	Cross sectional study	North America	36472595
Howard et al.	JAMA	Excess body weight [Exposure]	Epi O	Cohort	North America	36378216
Aiken et al.	JAMA		Epi O	DB analysis	North America	36318139
Henninger et al.	JAMA		Review	Systematic review	NA	36166040
Pineles et al.	JAMA		Epi O	DB analysis	North America	36318140
Tai et al.	JAMA		Epi O	Cohort	North America	35653123
Gupta, Pagán	JAMA		Epi O	DB analysis	North America	36573986
Patel et al.	JAMA	Illicit drugs [Exposure]	Epi O	DB analysis	North America	35191936
Xie et al.	BMJ	Smoking; Excess body weight; Physical activity [Exposure]	Epi O	DB analysis	Multicontinent	36740855
Shelmerdine et al.	BMJ		Other	Methodological study	UK	36543352
Ueda et al.	BMJ	Alcohol [Exposure]	Epi O	Cohort	Europe (UK excluded)	36543350
Gaesser et al.	BMJ	Physical activity [Exposure]	Epi I	Controlled trial	North America	36543338
Saul et al.	BMJ	Illicit drugs [Burden]	Other	Other	UK	36460314
McIlroy et al.	BMJ		Epi O	Cohort	North America	36450405
Diguisto et al.	BMJ		Epi O	DB analysis	UK & Other European countries	36384872
Jardine et al.	BMJ		Epi I	RCT	Multicontinent	36384746
Mohan et al.	BMJ		Other	Other	NA	36302551
Ross et al.	BMJ		Other	Other	NA	36302544
Newby et al.	BMJ		Other	Other	NA	36302540
Hovaguimian, Roth	BMJ		Review	Review	NA	36216384
Saul et al.	BMJ		Review	Systematic review	NA	36113881
Theilmann et al.	BMJ	Smoking [Burden]	Epi O	Cross sectional study	Multicontinent	36041745
Cro et al.	BMJ		Review	Systematic review	NA	35998928
Badell et al.	BMJ		Review	Review	NA	35948352
Gates et al.	BMJ		Other	Methodological study	Multicontinent	35944924
Saul et al.	BMJ	Physical activity [Exposure]	Epi I	RCT	UK	35905985
Saul et al.	BMJ		Other	Other	UK	35868651
McKeown et al.	BMJ		Other	Other	NA	35858693
Holgersson et al.	BMJ		Review	Systematic review	NA	35820685
Melnick et al.	BMJ	Illicit drugs [Outcome]	Epi I	RCT	North America	35760423
Bisson et al.	BMJ		Epi I	RCT	UK	35710124
Lee et al.	BMJ		Review	Systematic review	NA	35697365
Lu et al.	BMJ		Review	Review	NA	35613721
Hao et al.	BMJ		Other	Methodological study	NA	35508320
Luijken et al.	BMJ		Review	Review	Europe (UK excluded)	35504648
Cox, Fritz	BMJ		Other	Other	NA	35477529
Saul et al.	BMJ		Epi O	Cohort	UK	35459717
Sawalha, Maddocks	BMJ		Review	Review	NA	35443983
Lu et al.	BMJ		Epi I	RCT	Multicontinent	35440464
Avery et al.	BMJ		Review	Systematic review	NA	35379650
Smith et al.	BMJ		Epi O	Cohort	Oceania	35354610

Authors	Journal	Risk factor(s) [role]	Study Type	Study Design[a]	Geographic zone	PMID
Bénard-Laribière et al.	BMJ		Epi O	Case-control study	Europe (UK excluded)	35321876
Newman	BMJ		Other	Other	NA	35296503
Surkalim et al.	BMJ		Review	Systematic review	NA	35140066
Saul et al.	BMJ		Epi I	Controlled trial	UK	35135782
Ben Hassen et al.	BMJ		Epi O	Cohort	UK	35110302
Larsen et al.	BMJ	Physical activity [Exposure]	Review	Systematic review	NA	35082116
Buse et al.	BMJ		Other	Other	NA	37462013
Aybek, Perez	BMJ		Review	Review	NA	35074803
Chawla et al.	BMJ		Review	Systematic review	NA	35045991
Costa et al.	BMJ		Epi I	RCT	UK	35045969
Willis, de St Maurice	BMJ		Other	Other	NA	35022224
Weatherald et al.	BMJ		Review	Systematic review	NA	36740866
Castelli et al.	BMJ		Epi O	Case-control study	Other American countries	36450402
Tan et al.	BMJ		Review	Systematic review	NA	35896188
Hulme et al.	BMJ		Epi O	Cohort	UK	35858680
Hughes et al.	BMJ		Other	Methodological study	UK	35477524
Fralick et al.	BMJ		Epi I	RCT	North America	35321918
Li et al.	BMJ		Epi O	Cohort	UK & Other European countries	35296468
Fabiani et al.	BMJ		Epi O	Cohort	Europe (UK excluded)	35144968
Riley et al.	BMJ		Other	Other	NA	36593578
Ageno et al.	BMJ		Epi I	RCT	Europe (UK excluded)	36520715
Bonaccio et al.	BMJ		Epi O	Cohort	Europe (UK excluded)	36450651
Candal-Pedreira et al.	BMJ		Epi O	Cross sectional study	North America	36442874
Dixon-Woods	BMJ		Other	Other	NA	36400438
Zheng et al.	BMJ		Epi O	Cohort	UK	36384890
Briggs et al.	BMJ		Epi O	Cohort	UK	36351667
Pereira et al.	BMJ		Review	Systematic review	NA	36333100
Futier et al.	BMJ		Epi I	RCT	Europe (UK excluded)	36328372
Pradhan et al.	BMJ		Epi O	Cohort	UK	36318979
Blom et al.	BMJ		Epi I	RCT	UK & Other European countries	36316046
Vijayaraghavan et al.	BMJ		Other	Other	NA	36302546
Blum et al.	BMJ		Other	Other	NA	36302526
Taneja et al.	BMJ	Smoking; Alcohol; Excess body weight; Physical activity [Recommendation]	Epi I	RCT	Asia	36288808
Goodney et al.	BMJ		Epi O	Cohort	North America	36283705
Weintraub et al.	BMJ		Review	Review	NA	36280256
Wang et al.	BMJ	Excess body weight [Outcome]	Epi O	Cohort	North America	36198411
Saul et al.	BMJ		Other	Other	UK	36195322
Myers et al.	BMJ		Other	Other	NA	36175018
Hägglund et al.	BMJ		Other	Other	NA	36175012
Auer et al.	BMJ		Epi I	RCT	North America	36100263
Stone et al.	BMJ		Epi I	RCT	North America	35918097
Horne et al.	BMJ		Epi O	Cohort	UK	35858698
Saul et al.	BMJ		Epi O	Case-control study	UK	35787513
Saul et al.	BMJ		Other	Other	UK	35764321
Liang et al.	BMJ		Epi O	individual participant	Multicontinent	35732311

Authors	Journal	Risk factor(s) [role]	Study Type	Study Design[a]	Geographic zone	PMID
				Meta-analysis		
Saul et al.	BMJ		Other	Other	UK	35714978
Al-Lamee et al.	BMJ		Review	Review	NA	35697356
Goostrey, Muehlschlegel	BMJ		Review	Review	NA	35696329
Rebolj et al.	BMJ		Epi O	Cohort	UK	35640960
Ye et al.	BMJ		Review	Systematic review	NA	35613728
Kawahara et al.	BMJ		Epi I	RCT	Asia	35613725
Šumilo et al.	BMJ		Epi O	Cohort	UK	35580876
Khan et al.	BMJ		Review	Systematic review	NA	35508321
Cram et al.	BMJ		Epi O	Cohort	Multicontinent	35508312
Baum et al.	BMJ		Other	Other	NA	35504651
Nana et al.	BMJ		Review	Review	NA	35473709
Paskins et al.	BMJ		Epi I	RCT	UK	35387783
Saul et al.	BMJ		Other	Qualitative study	UK	35379673
Potter et al.	BMJ		Epi O	DB analysis	North America	35354556
Schindler, Burish	BMJ		Review	Review	NA	35296510
Harding et al.	BMJ		Epi I	RCT	UK	35264408
Saul et al.	BMJ		Epi O	Case-control study	UK	35260385
McCracken et al.	BMJ		Review	Review	NA	35228207
Lu et al.	BMJ		Other	Other	NA	35217525
Li et al.	BMJ		Other	Economic study	NA	35217521
Fei et al.	BMJ		Other	Methodological study	NA	35217507
Zhang et al.	BMJ		Other	Other	NA	35217506
NA	BMJ	Smoking [Outcome]	Epi I	RCT	Europe (UK excluded)	35193871
Gadjradj et al.	BMJ		Epi I	RCT	Europe (UK excluded)	35190388
Chan et al.	BMJ		Epi O	Cohort	North America	35173019
Saul et al.	BMJ		Epi O	Cohort	UK	35168943
Saul et al.	BMJ		Other	Other	UK	35082137
Husereau et al.	BMJ		Other	Economic study	UK	35017145
Peter et al.	BMJ		Epi O	Cross sectional study	Europe (UK excluded)	36229057
Adams et al.	BMJ		Epi O	Case-control study	North America	36220174
Schuit et al.	BMJ		Epi O	Cross sectional study	Europe (UK excluded)	36104069
Pillay et al.	BMJ		Review	Review	NA	35830976
Gazit et al.	BMJ		Epi O	Case-control study	Asia	35609888
Ayoubkhani et al.	BMJ		Epi O	Cohort	UK	35584816
Lauring et al.	BMJ		Epi O	Case-control study	North America	35264324
Lee et al.	BMJ		Review	Systematic review	NA	35236664
Kamran et al.	BMJ		Epi O	Cohort	North America	35177406
Xie et al.	BMJ		Epi O	Cohort	North America	35172971
Kadambari et al.	BMJ		Epi O	DB analysis	UK	35022215
Santer et al.	BMJ		Epi I	RCT	UK	36740888
Jones, Francis	BMJ		Other	Other	UK	36535701
Schuurman et al.	BMJ		Epi O	Cross sectional study	Europe (UK excluded)	36535672
Rahangdale et al.	BMJ		Review	Review	NA	36521855
Chovanec, Cheng	BMJ		Review	Review	NA	36442868
Saul et al.	BMJ		Review	Systematic review	NA	36400449
Horne, Missmer	BMJ		Review	Review	NA	36375827

Authors	Journal	Risk factor(s) [role]	Study Type	Study Design[a]	Geographic zone	PMID
Archer et al.	BMJ		Epi O	Cohort	UK	36347531
Ward et al.	BMJ		Epi O	DB analysis	UK	36323407
McGushin et al.	BMJ		Other	Other	NA	36302556
NA	BMJ		Epi I	RCT	Europe (UK excluded)	36261169
Tappin et al.	BMJ	Smoking [Outcome]	Epi I	RCT	UK	36261162
Dhodapkar et al.	BMJ		Epi O	Cross sectional study	North America	36198428
Hendriks et al.	BMJ		Other	Other	NA	36175028
Guinto et al.	BMJ		Other	Other	NA	36175023
Hudda et al.	BMJ	Excess body weight [Outcome]	Epi O	individual participant Meta-analysis	UK	36130780
Bundred et al.	BMJ		Review	Systematic review	NA	36130770
Xie et al.	BMJ		Review	Systematic review	NA	36130740
Hodkinson et al.	BMJ		Review	Systematic review	NA	36104064
Edwardson et al.	BMJ	Physical activity [Outcome]	Epi I	RCT	UK	35977732
Rees et al.	BMJ		Epi O	Cohort	UK	35938625
Kechagias et al.	BMJ		Review	Systematic review	NA	35922074
Alderwick et al.	BMJ		Other	Other	UK	35788447
Eck et al.	BMJ		Review	Systematic review	NA	35788047
He et al.	BMJ		Review	Systematic review	NA	35764326
Ibrahim et al.	BMJ		Epi O	Cross sectional study	North America	35738660
Xie, Zhou	BMJ		Epi O	DB analysis	North America	35732297
Warraich et al.	BMJ		Epi O	DB analysis	North America	35672032
Saul et al.	BMJ		Epi I	RCT	UK	35636772
Saul et al.	BMJ		Epi O	Cohort	UK	35605994
Phillips et al.	BMJ		Other	Other	UK	35577357
Bernabei et al.	BMJ	Physical activity [Outcome]	Epi I	RCT	Europe (UK excluded)	35545258
Kotwal et al.	BMJ		Epi I	RCT	Oceania	35414532
Ho et al.	BMJ		Review	Systematic review	NA	35354560
Wade et al.	BMJ		Other	Other	NA	35351684
Saul et al.	BMJ		Review	Systematic review	NA	35346973
Hopkins, Narasimhan	BMJ		Other	Other	NA	35331989
Hodkinson et al.	BMJ		Review	Systematic review	NA	35331984
Chaudhry et al.	BMJ		Review	Systematic review	NA	35232772
Zeng et al.	BMJ		Other	Methodological study	NA	35217581
Haseler, Haseler	BMJ	Physical activity [Burden]	Review	Review	NA	35197324
Care et al.	BMJ		Review	Systematic review	NA	35168930
Mac Grory et al.	BMJ		Review	Review	North America	35140114
He et al.	BMJ		Epi I	RCT	Asia	35140061
Homer et al.	BMJ		Other	Methodological study	NA	35131744
Dhaliwal et al.	BMJ		Other	Other	NA	35086910
Brush et al.	BMJ		Review	Review	NA	34987062
Rouyard et al.	BMJ	Smoking; Alcohol; Excess body weight; Physical activity [Recommendation]	Other	Other	NA	34983778
Zhang et al.	BMJ		Epi O	Cohort	UK	36418047
Jolliffe et al.	BMJ		Epi I	RCT	UK	36215226
Brunvoll et al.	BMJ		Epi I	RCT	Europe (UK excluded)	36215222

Authors	Journal	Risk factor(s) [role]	Study Type	Study Design[a]	Geographic zone	PMID
Ferdinands et al.	BMJ		Epi O	Case-control study	North America	36191948
Pople et al.	BMJ		Epi O	Cohort	UK	35858689
Andersson et al.	BMJ		Epi O	Cohort	Europe (UK excluded)	35831006
de Jong et al.	BMJ		Epi O	individual participant Meta-analysis	Multicontinent	35820692
Grewal et al.	BMJ		Epi O	Case-control study	North America	35793826
Duval et al.	BMJ		Review	Systematic review	NA	35768139
Au, Cheung	BMJ		Review	Systematic review	NA	35640925
Moneer et al.	BMJ		Review	Systematic review	NA	35537738
Suthar et al.	BMJ		Epi O	DB analysis	North America	35477670
Deeks et al.	BMJ		Epi O	DB analysis	UK	35197270
Cohen et al.	BMJ		Epi O	Cohort	North America	35140117
Liu et al.	BMJ		Review	Review	NA	36946547
Debras et al.	BMJ		Epi O	Cohort	Europe (UK excluded)	36638072
Kotecha et al.	BMJ		Other	Methodological study	Europe (UK excluded)	36562446
Paytubi et al.	BMJ		Epi O	Cross sectional study	Europe (UK excluded)	36543351
Biddle et al.	BMJ	Physical activity [Outcome]	Epi I	RCT	UK	36535688
Stein et al.	BMJ		Other	Other	NA	36535671
Schroter et al.	BMJ		Epi O	Cohort	UK	36517041
NA	BMJ		Epi I	RCT	Europe (UK excluded)	36455932
Nguyen et al.	BMJ		Review	Review	NA	36414269
Saul et al.	BMJ		Other	Other	UK	36368720
Baltag et al.	BMJ		Other	Other	NA	36302523
Li et al.	BMJ		Epi O	Cohort	Multicontinent	36288813
Saul et al.	BMJ	Excess body weight [Exposure]	Epi O	Cohort	UK	36283709
Huang et al.	BMJ		Epi O	cohort	Europe (UK excluded)	36261141
Atwoli et al.	BMJ		Other	Other	Africa	36257789
Aggarwal et al.	BMJ		Other	Other	NA	36216385
Wang et al.	BMJ		Epi O	Cohort	North America	36195324
T'Sjoen, Motmans	BMJ		Other	Other	NA	36191953
Deng et al.	BMJ		Epi O	Cohort	North America	36191949
Barry et al.	BMJ		Other	Other	NA	36175019
Free et al.	BMJ		Epi I	RCT	UK	36170988
Okoshi et al.	BMJ		Epi O	Cohort	Asia	36170985
Yang et al.	BMJ	Smoking; Alcohol; Excess body weight; Physical activity [Exposure]	Epi O	Cohort	North America	36130782
Patel et al.	BMJ		Other	Case series	UK	35902115
Safiri et al.	BMJ	Smoking [Burden]	Epi O	DB analysis	Multicontinent	35896191
Jonas	BMJ	Smoking [Exposure]	Review	Review	NA	35851281
Saul et al.	BMJ		Epi O	individual participant Meta-analysis	UK	35817423
Valbuena et al.	BMJ		Epi O	Cohort	North America	35793817
Williams et al.	BMJ		Other	Qualitative study	NA	35688481
Saul et al.	BMJ		Epi I	Controlled trial	UK	35688460
Madigan et al.	BMJ	Excess body weight [Outcome]	Review	Systematic review	NA	35636762

Authors	Journal	Risk factor(s) [role]	Study Type	Study Design[a]	Geographic zone	PMID
Vasey et al.	BMJ		Other	Methodological study	NA	35584845
Xu et al.	BMJ		Other	Methodological study	NA	35537752
Dhana et al.	BMJ	Smoking; Alcohol; Excess body weight; Physical activity [Exposure]	Epi O	Cohort	North America	35418416
Zhang et al.	BMJ		Other	Methodological study	NA	35354583
Yamey et al.	BMJ		Other	Other	NA	35331982
Teshome et al.	BMJ		Epi O	Cohort	North America	35318190
Saul et al.	BMJ		Other	Other	UK	35302048
NA	BMJ	Smoking; Alcohol; Excess body weight; Physical activity [Burden]	Epi O	DB analysis	Multicontinent	35273014
Marcucci et al.	BMJ		Review	Systematic review	NA	35264372
Walsh et al.	BMJ		Review	Systematic review	NA	35217545
Kettle et al.	BMJ	Physical activity [Recommendation]	Review	Systematic review	NA	35197242
Gopinathan, Buse	BMJ		Other	Other	NA	35135754
Abbar et al.	BMJ		Epi I	RCT	Europe (UK excluded)	35110300
Saul et al.	BMJ	Excess body weight [Exposure]	Epi O	Cohort	UK	35110298
Hahn et al.	BMJ		Epi I	RCT	North America	35082139
Labonté	BMJ		Other	Economic study	NA	35078773
Coombes, Davies	BMJ		Other	Other	NA	35045953
Jayedi et al.	BMJ	Excess body weight [Exposure]	Review	Systematic review	NA	35042741
Powell et al.	BMJ		Other	Other	UK	35042720
NA	BMJ		Epi I	RCT	Asia	34996756
Gasbjerg et al.	BMJ		Epi I	RCT	Europe (UK excluded)	34983775
Fell et al.	BMJ		Epi O	Cohort	North America	35977737
Ward et al.	BMJ		Epi O	Cohort	UK	35918098
Hitchings et al.	BMJ		Epi O	Case-control study	Other American countries	35697361
Urner et al.	BMJ		Epi O	Cohort	Multicontinent	35508314
Kildegaard et al.	BMJ		Epi O	Cohort	Europe (UK excluded)	35410884
Katsoularis et al.	BMJ		Epi O	Cohort	Europe (UK excluded)	35387772
Allotey et al.	BMJ		Review	Systematic review	NA	35296519
Magnusson et al.	BMJ		Epi O	DB analysis	Europe (UK excluded)	35039315
Tweed	Lancet		Epi O	Cross sectional study	UK	36930031
Torresin et al.	Lancet		Epi O	Cross sectional study	UK	36930029
Burns et al.	Lancet		Epi O	Cohort	UK	36929995
Gray et al.	Lancet		Epi O	Cross sectional study	UK	36929988
Huf et al.	Lancet		Epi O	Cohort	UK	36929986
Geismar et al.	Lancet		Epi O	Cohort	UK	36929985
Manca et al.	Lancet	Alcohol [Burden]	Epi O	DB analysis	UK	36929952
Cicinelli et al.	Lancet		Review	Review	NA	36565712
Adams et al.	Lancet		Review	Review	NA	36563698
Forrest et al.	Lancet		Epi O	Cohort	North America	36563696
Frenk et al.	Lancet		Review	Review	NA	36522209
Brennan et al.	Lancet		Epi I	RCT	UK	36522207
Abubakar et al.	Lancet		Other	Other	NA	36502851
Devakumar et al.	Lancet		Review	Review	NA	36502848

Authors	Journal	Risk factor(s) [role]	Study Type	Study Design[a]	Geographic zone	PMID
Thurber et al.	Lancet		Epi O	Cross sectional study	Oceania	36502846
Sheikh et al.	Lancet		Epi O	DB analysis	Multicontinent	36502843
Mitjà et al.	Lancet		Review	Review	NA	36403582
Vrselja et al.	Lancet		Epi I	Phase 1/2 study	Multicontinent	36366886
Schlaich et al.	Lancet		Epi I	RCT	Multicontinent	36356632
Urva et al.	Lancet	Excess body weight [Outcome]	Epi I	Phase 1/2 study	North America	36354040
Bistervels et al.	Lancet		Epi I	RCT	Multicontinent	36354038
Li et al.	Lancet		Epi I	RCT	Asia	36351459
Kalra et al.	Lancet		Epi I	RCT	UK	36347265
NA	Lancet		Epi I	RCT	North America	36343653
Myers et al.	Lancet	Alcohol [Outcome]	Epi I	RCT	Africa	36244383
Klapsa et al.	Lancet		Chemical/toxicological, in vivo, in vitro	Chemical study	UK	36243024
Simon-Kumar et al.	Lancet		Epi O	DB analysis	Oceania	36183729
Noseworthy et al.	Lancet		Epi I	Controlled trial	North America	36179758
Ni et al.	Lancet		Epi O	Cross sectional study	Asia	36154677
Donnez et al.	Lancet		Epi I	RCT	North America	36116480
Perlis et al.	Lancet		Review	Review	NA	36115372
Pérez-Carbonell et al.	Lancet		Review	Review	NA	36115367
Kim et al.	Lancet		Epi I	RCT	North America	36108657
Livingstone et al.	Lancet		Epi I	RCT	Europe (UK excluded)	36099927
NA	Lancet		Epi O	individual participant Meta-analysis	Multicontinent	36049498
Wenzl et al.	Lancet		Epi O	Cohort	UK & Other European countries	36049493
Uyeki et al.	Lancet		Review	Review	NA	36030813
Chihara et al.	Lancet		Epi O	DB analysis	North America	35964611
Chua et al.	Lancet		Epi I	RCT	Multicontinent	35934006
Deodhar et al.	Lancet		Epi I	RCT	Multicontinent	35908570
Reich et al.	Lancet		Epi I	RCT	Multicontinent	35871814
Kim et al.	Lancet		Epi I	RCT	Asia	35863366
NA	Lancet	Alcohol [Burden]	Review	Systematic review	NA	35843246
De Crescenzo et al.	Lancet		Review	Systematic review	NA	35843245
Fischer et al.	Lancet		Epi I	RCT	Multicontinent	35810756
Sacco et al.	Lancet		Epi O	Cohort	Europe (UK excluded)	35780801
Maercker et al.	Lancet		Review	Review	NA	35780794
Perry et al.	Lancet		Epi I	RCT	UK	35780790
Hyder et al.	Lancet		Review	Review	NA	35779552
Vecino-Ortiz et al.	Lancet		Review	Systematic review	NA	35779550
Fiore et al.	Lancet		Review	Systematic review	NA	35717988
Li et al.	Lancet		Epi I	Phase 1/2 study	UK	35691324
Sands et al.	Lancet		Epi I	RCT	Multicontinent	35691323
Wong et al.	Lancet		Epi O	Cohort	North America	35691322
Kattamis et al.	Lancet		Review	Review	NA	35691301
NA	Lancet		Epi O	DB analysis	Multicontinent	35617980
Christenson et al.	Lancet	Smoking [Burden]	Review	Review	NA	35533707
Smits et al.	Lancet		Epi I	RCT	Europe (UK excluded)	35490691
Black et al.	Lancet		Review	Review	Africa	35489357
Sethi et al.	Lancet		Review	Review	NA	35461559
Saji et al.	Lancet		Epi I	RCT	Asia	35461558
Tonorezos et al.	Lancet		Review	Review	NA	35430023

Authors	Journal	Risk factor(s) [role]	Study Type	Study Design[a]	Geographic zone	PMID
Jefford et al.	Lancet		Review	Review	NA	35430022
Kanungo et al.	Lancet		Review	Review	NA	35397865
Mahfoud et al.	Lancet		Epi I	RCT	Multicontinent	35390320
Piccini et al.	Lancet		Epi I	Phase 1/2 study	Multicontinent	35385695
Ezekowitz et al.	Lancet		Epi I	RCT	Multicontinent	35381194
Bergmark et al.	Lancet		Review	Review	NA	35367005
Mosha et al.	Lancet		Epi I	RCT	Africa	35339225
NA	Lancet		Review	Systematic review	NA	35279232
McGarvey et al.	Lancet		Epi I	RCT	Multicontinent	35248186
Shah et al.	Lancet		Epi I	RCT	Multicontinent	35120593
NA	Lancet		Epi I	RCT	Multicontinent	35093205
Heier et al.	Lancet		Epi I	RCT	Multicontinent	35085502
Caeymaex et al.	Lancet		Epi I	RCT	Europe (UK excluded)	35065786
Ramacciotti et al.	Lancet		Epi I	RCT	Other American countries	34921756
Moon et al.	Lancet		Review	Review	NA	34902308
Lingvay et al.	Lancet	Excess body weight [Outcome]	Review	Review	NA	34600604
Scott et al.	Lancet		Other	Qualitative study	UK	36930026
Rosenthal et al.	Lancet		Epi O	Cross sectional study	UK	36930023
Makanjuola et al.	Lancet		Epi O	Cohort	UK	36930005
Keeble et al.	Lancet		Epi O	Cohort	UK	36930000
Curtin et al.	Lancet		Other	Qualitative study	UK	36929978
Whitehead, Wells	Lancet		Epi O	Cross sectional study	UK	36929953
Griffiths et al.	Lancet		Epi O	Cohort	UK	36929951
Butler et al.	Lancet		Epi I	RCT	UK	36566761
Marwaha et al.	Lancet		Review	Review	NA	36535295
Mok et al.	Lancet		Epi I	RCT	Asia	36522208
Guttman-Yassky et al.	Lancet		Epi I	Phase 1/2 study	Multicontinent	36509097
Selvarajah et al.	Lancet		Review	Review	NA	36502849
Kramer et al.	Lancet	Excess body weight; Physical activity [Burden]	Review	Systematic review	NA	36502845
Auer et al.	Lancet		Review	Review	NA	36502822
Zaman et al.	Lancet		Epi I	Phase 1/2 study	Asia	36495882
Hurvitz et al.	Lancet		Epi I	RCT	Multicontinent	36495879
Saito et al.	Lancet		Review	Systematic review	NA	36442488
Chen et al.	Lancet	Smoking; Alcohol; Excess body weight; Physical activity [Recommendation]	Review	Review	Asia	36423650
Gostin et al.	Lancet		Other	Other	NA	36403583
Ahmad et al.	Lancet		Review	Review	NA	36332637
NA	Lancet		Epi I	RCT	Multicontinent	36328045
NA	Lancet		Epi O	DB analysis	Multicontinent	36328042
Mackenzie et al.	Lancet		Epi I	RCT	UK	36216006
Meyer et al.	Lancet		Review	Review	NA	36115370
Walli-Attaei et al.	Lancet	Smoking; Excess body weight [Exposure]	Epi O	Cohort	North America	36088949
Pitcher et al.	Lancet		Review	Systematic review	NA	36049495
Vaduganathan et al.	Lancet		Review	Systematic review	NA	36041474
Endler et al.	Lancet		Epi I	RCT	Africa	36030811
Tesfaye et al.	Lancet		Epi I	RCT	UK	36007534
Jackson et al.	Lancet		Epi I	RCT	North America	35964610
Mitchell et al.	Lancet		Epi I	RCT	Multicontinent	35810757
Carlson, Phelan	Lancet		Review	Review	NA	35810748
Dalziel et al.	Lancet		Review	Review	NA	35785792
Menon et al.	Lancet		Epi I	RCT	North America	35779553
Giudice et al.	Lancet		Epi I	RCT	Multicontinent	35717987

Authors	Journal	Risk factor(s) [role]	Study Type	Study Design[a]	Geographic zone	PMID
Shiffman, Shawar	Lancet		Review	Review	NA	35594874
Pollack et al.	Lancet		Epi I	RCT	Multicontinent	35569466
Knipe et al.	Lancet		Review	Review	NA	35512727
Kruk et al.	Lancet		Review	Review	NA	35489361
Victora et al.	Lancet		Epi O	DB analysis	Multicontinent	35489358
Trutnovsky et al.	Lancet		Epi I	RCT	Europe (UK excluded)	35483400
NA	Lancet		Epi O	DB analysis	North America	35405084
Crosbie et al.	Lancet	Excess body weight [Exposure]	Review	Review	NA	35397864
NA	Lancet		Epi O	DB analysis	Asia	35397236
Sessler et al.	Lancet		Epi I	RCT	Multicontinent	35390321
Luke et al.	Lancet		Epi I	RCT	Multicontinent	35367007
Winter, Moss	Lancet		Review	Review	NA	35367004
Khobragade et al.	Lancet		Epi I	RCT	Asia	35367003
Bekker et al.	Lancet		Epi I	RCT	Africa	35305740
Nyberg et al.	Lancet		Epi O	Cohort	UK	35305296
Yen et al.	Lancet		Other	Case series	Asia	35279259
NA	Lancet		Epi O	DB analysis	Multicontinent	35219376
Feikin et al.	Lancet		Review	Systematic review	NA	35202601
Sardinha et al.	Lancet		Epi O	DB analysis	UK	35182472
Nordström et al.	Lancet		Epi O	Cohort	Europe (UK excluded)	35131043
Gershenson et al.	Lancet		Epi I	RCT	North America & UK	35123694
Tromp et al.	Lancet		Epi O	Cohort	Multicontinent	35101175
Wykoff et al.	Lancet		Epi I	RCT	North America	35085503
Costa Clemens et al.	Lancet		Epi I	RCT	Other American countries	35074136
NA	Lancet		Epi O	DB analysis	Multicontinent	35065702
Stuart et al.	Lancet		Epi I	RCT	UK	34883053
Norris et al.	Lancet	Excess body weight [Recommendation]	Other	Other	NA	34856190
Qi et al.	Lancet		Epi O	DB analysis	UK	36930016
Niranjan et al.	Lancet	Smoking; Alcohol; Excess body weight; Physical activity [Recommendation]	Epi I	Controlled trial	Europe (UK excluded)	36930014
Dawes et al.	Lancet		Other	Qualitative study	UK	36929979
Bright et al.	Lancet		Epi O	Cohort	UK	36929968
Bernard et al.	Lancet		Epi O	Cohort	UK	36929963
Andreae et al.	Lancet		Review	Systematic review	NA	36929959
Mahdi et al.	Lancet		Epi I	RCT	UK	36929955
Parnham et al.	Lancet		Epi O	Cross sectional study	UK	36929954
Birgand et al.	Lancet		Review	Review	NA	36528378
Krystal et al.	Lancet		Epi I	RCT	North America	36528376
Butterfield et al.	Lancet		Epi O	Cohort	North America	36502844
Merola et al.	Lancet		Epi I	RCT	Multicontinent	36495881
McInnes et al.	Lancet		Epi I	RCT	Multicontinent	36493791
NA	Lancet		Epi O	DB analysis	Multicontinent	36423648
Thornhill et al.	Lancet		Other	Case series	Multicontinent	36403584
NA	Lancet		Review	Systematic review	NA	36351458
Hodgetts Morton et al.	Lancet		Epi I	RCT	UK	36273481
Agrawal et al.	Lancet		Epi O	Cohort	UK	36244382
Wong et al.	Lancet		Epi O	Cohort	Asia	36216007
Compérat et al.	Lancet		Review	Review	NA	36174585
Bhatt et al.	Lancet		Epi I	RCT	North America	36130612
Paller et al.	Lancet		Epi I	RCT	Multicontinent	36116481

Authors	Journal	Risk factor(s) [role]	Study Type	Study Design[a]	Geographic zone	PMID
Feldman et al.	Lancet		Review	Review	NA	36116464
Bos, Ware	Lancet		Review	Review	NA	36070787
Beard et al.	Lancet		Epi I	RCT	UK	35988569
Schmitz et al.	Lancet		Epi I	RCT	Europe (UK excluded)	35988568
NA	Lancet	Smoking; Alcohol; Excess body weight; Physical activity [Burden]	Epi O	DB analysis	Multicontinent	35988567
Claassen, Park	Lancet	Smoking [Exposure]	Review	Review	NA	35985353
Tarín-Vicente et al.	Lancet		Epi O	Cohort	Europe (UK excluded)	35952705
Thapar et al.	Lancet		Review	Review	NA	35940184
NA	Lancet		Epi I	RCT	UK	35908569
Abu Dayyeh et al.	Lancet	Excess body weight [Outcome]	Epi I	RCT	North America	35908555
Haakenstad et al.	Lancet		Epi O	DB analysis	Multicontinent	35871816
Krijbolder et al.	Lancet		Epi I	RCT	Europe (UK excluded)	35871815
Stewart et al.	Lancet		Review	Review	NA	35868329
Wolf et al.	Lancet		Review	Systematic review	NA	35780792
Razzak et al.	Lancet		Review	Review	NA	35779549
Reed et al.	Lancet		Epi O	cohort	North America	35658995
Danese et al.	Lancet		Epi I	RCT	Multicontinent	35644166
D'Haens et al.	Lancet		Epi I	RCT	Multicontinent	35644154
Kickbusch, Liu	Lancet		Review	Systematic review	NA	35594877
NA	Lancet		Review	Systematic review	NA	35512728
Vaivada et al.	Lancet		Review	Review	NA	35489360
Metcalfe et al.	Lancet		Epi I	RCT	UK	35461618
Nguyen et al.	Lancet		Epi I	RCT	Asia	35427481
Fizazi et al.	Lancet		Epi I	RCT	Europe (UK excluded)	35405085
Flor et al.	Lancet		Review	Review	NA	35247311
Schneider-Thoma et al.	Lancet		Review	Systematic review	NA	35219395
Craig et al.	Lancet		Epi I	Phase 1/2 study	Multicontinent	35219377
NA	Lancet		Epi O	DB analysis	Multicontinent	35120592
Jauhar et al.	Lancet		Review	Review	NA	35093231
Fumagalli et al.	Lancet		Epi I	Phase 1/2 study	Europe (UK excluded)	35065785
Bravo et al.	Lancet		Epi I	RCT	Multicontinent	35065705
Kentish-Barnes et al.	Lancet		Epi I	RCT	Europe (UK excluded)	35065008
Attard et al.	Lancet		Review	Systematic review	NA	34953525
Bowman et al.	Lancet		Epi I	Phase 1/2 study	Multicontinent	34861168
Neufeld et al.	Lancet		Review	Review	NA	34856191
NA	Lancet		Epi I	RCT	Multicontinent	34800427
McNamara et al.	Lancet		Epi O	DB analysis	North America	34741818
Dowrick et al.	Lancet		Other	Qualitative study	UK	36930035
McKinlay et al.	Lancet		Other	Qualitative study	UK	36930010
Bach-Mortensen et al.	Lancet		Epi O	Cohort	UK	36929956
Shannon et al.	Lancet		Review	Review	NA	36502850
Volkmann et al.	Lancet		Review	Review	NA	36442487
Thompson et al.	Lancet		Epi I	RCT	Multicontinent	36442484
Weatherald et al.	Lancet		Review	Review	NA	36436527
Goodall et al.	Lancet		Epi I	RCT	Multicontinent	36368336
Jones et al.	Lancet		Epi O	individual participant Meta-analysis	Oceania	36366885
Yang et al.	Lancet		Epi I	RCT	Asia	36341753
Hawkey et al.	Lancet		Epi I	RCT	UK	36335970
Michael et al.	Lancet		Review	Review	NA	36272423
NA	Lancet		Epi I	RCT	Multicontinent	36244384

Authors	Journal	Risk factor(s) [role]	Study Type	Study Design[a]	Geographic zone	PMID
Mackenzie et al.	Lancet		Epi I	RCT	UK	36240838
Luo et al.	Lancet		Review	Systematic review	NA	36116479
Pal et al.	Lancet		Epi I	RCT	Multicontinent	36099926
Vogel et al.	Lancet	Alcohol [Exposure]	Review	Review	NA	36084663
Gorman et al.	Lancet		Review	Review	NA	36070788
Shoamanesh et al.	Lancet		Epi I	Phase 1/2 study	Europe (UK excluded)	36063821
Conrad et al.	Lancet		Epi O	DB analysis	UK	36041475
Kuo et al.	Lancet		Epi I	Phase 1/2 study	North America & UK	35964609
Wijsenbeek et al.	Lancet		Review	Review	NA	35964592
Ballering et al.	Lancet		Epi O	Cohort	Europe (UK excluded)	35934007
Skjerven et al.	Lancet		Epi I	RCT	Europe (UK excluded)	35753340
NA	Lancet		Epi O	DB analysis	North America	35717994
Kamdar et al.	Lancet		Epi I	RCT	Multicontinent	35717989
Catassi et al.	Lancet		Review	Review	NA	35691302
Ferrante et al.	Lancet		Epi I	RCT	Multicontinent	35644155
Li et al.	Lancet		Review	Review	NA	35598608
Ho et al.	Lancet		Review	Review	NA	35594878
Delgado-Lista et al.	Lancet		Epi I	RCT	Europe (UK excluded)	35525255
Sun et al.	Lancet	[Recommendation]	Epi I	RCT	Asia	35500594
Emery et al.	Lancet	Smoking; Alcohol; Excess body weight; Physical activity [Exposure]	Review	Review	NA	35430021
Menni et al.	Lancet		Epi O	Cohort	UK	35397851
Delany-Moretlwe et al.	Lancet		Epi I	RCT	Africa	35378077
NA	Lancet	Smoking; Alcohol; Excess body weight; Physical activity [Recommendation]	Review	Review	NA	35339227
Edqvist et al.	Lancet		Epi I	RCT	Europe (UK excluded)	35303474
Rearte et al.	Lancet		Epi O	Case-control study	Other American countries	35303473
Angell et al.	Lancet		Epi O	DB analysis	Africa	35303469
NA	Lancet		Epi O	DB analysis	Africa	35294898
Reid, Billington	Lancet		Review	Review	NA	35279261
McDonald et al.	Lancet		Epi I	Phase 1/2 study	North America	35279258
van der Steen et al.	Lancet		Epi I	RCT	Europe (UK excluded)	35240044
NA	Lancet		Epi I	RCT	UK	35151397
Hübschen et al.	Lancet		Review	Review	NA	35093206
Jones et al.	Lancet		Epi I	RCT	North America	35065784
Wolter et al.	Lancet		Epi O	DB analysis	Africa	35065011
Halperin et al.	Lancet		Epi I	RCT	Multicontinent	34953526
Katikireddi et al.	Lancet		Epi O	Cohort	Multicontinent	34942103
Peeling et al.	Lancet		Review	Review	NA	34942102
Shi et al.	Lancet	Excess body weight [Outcome]	Review	Systematic review	NA	34895470
Hargreaves et al.	Lancet	Excess body weight; Physical activity [Recommendation]	Review	Review	NA	34856192
Jovin et al.	Lancet		Review	Systematic review	NA	34774198
Mebazaa et al.	Lancet		Epi I	RCT	Multicontinent	36356631
Herrington et al.	NEJM		Epi I	RCT	Multicontinent	36331190
van Dyck et al.	NEJM		Epi I	RCT	Multicontinent	36449413
Solomon et al.	NEJM		Epi I	RCT	Multicontinent	36027570
Weghuber et al.	NEJM	Excess body weight [Outcome]	Epi I	RCT	Multicontinent	36322838
Mullens et al.	NEJM		Epi I	RCT	Europe (UK excluded)	36027559

Authors	Journal	Risk factor(s) [role]	Study Type	Study Design[a]	Geographic zone	PMID
Ishani et al.	NEJM		Epi I	RCT	North America	36516076
Perera et al.	NEJM		Epi I	RCT	UK	36027563
Das Pradhan et al.	NEJM		Epi I	RCT	Multicontinent	36342113
Bhandari et al.	NEJM		Epi I	RCT	UK	36326117
Dickinson et al.	NEJM		Epi I	Phase 1/2 study	Multicontinent	36507690
Bretthauer et al.	NEJM		Epi I	RCT	Multicontinent	36214590
Lamontagne et al.	NEJM		Epi I	RCT	North America	35704292
Goodwin et al.	NEJM		Epi I	Phase 1/2 study	Multicontinent	36322843
Castellano et al.	NEJM		Epi I	RCT	Europe (UK excluded)	36018037
Farber et al.	NEJM		Epi I	RCT	Multicontinent	36342173
Connolly et al.	NEJM		Epi I	RCT	Multicontinent	36036525
Miller et al.	NEJM		Epi I	RCT	Multicontinent	36129998
Hodgson et al.	NEJM		Epi I	RCT	Oceania	36286256
Nathan et al.	NEJM		Epi I	RCT	North America	36129997
Cao et al.	NEJM		Epi I	RCT	Asia	36577095
Dellon et al.	NEJM		Epi I	RCT	Multicontinent	36546624
Griffith et al.	NEJM		Other	Case series	North America	35731912
Thornhill et al.	NEJM		Other	Case series	Multicontinent	35866746
Chalkias et al.	NEJM		Epi I	Phase 2-3 study	North America	36112399
Chari et al.	NEJM		Epi I	Phase 1/2 study	Multicontinent	36507686
Nathan et al.	NEJM		Epi I	RCT	North America	36129996
Rosmarin et al.	NEJM		Epi I	RCT	Multicontinent	36260792
Rohaan et al.	NEJM		Epi I	RCT	Europe (UK excluded)	36477031
Cheskes et al.	NEJM		Epi I	RCT	North America	36342151
Brown et al.	NEJM		Epi I	RCT	Europe (UK excluded)	36511784
Andersen-Ranberg et al.	NEJM		Epi I	RCT	UK & Other European countries	36286254
Andrade et al.	NEJM		Epi O	Cohort	North America	36342178
Kjaergaard et al.	NEJM		Epi I	RCT	Europe (UK excluded)	36027564
Yaeger et al.	NEJM		Epi I	Phase 1/2 study	North America	36546659
Freeman et al.	NEJM		Epi I	RCT	North America	36342143
Castellino et al.	NEJM		Epi I	RCT	North America	36322844
Thille et al.	NEJM		Epi I	RCT	Europe (UK excluded)	36286317
Yuen et al.	NEJM		Epi I	Phase 1/2 study	Multicontinent	36346079
O'Donoghue et al.	NEJM		Epi I	RCT	Multicontinent	36342163
Semler et al.	NEJM		Epi I	RCT	North America	36278971
Arbel et al.	NEJM		Epi O	Cohort	Asia	36001529
Jovin et al.	NEJM		Epi I	RCT	Asia	36239645
Schmidt et al.	NEJM		Epi I	RCT	Europe (UK excluded)	36027567
Kapadia et al.	NEJM		Epi I	RCT	Multicontinent	36121045
Sorensen et al.	NEJM		Epi I	RCT	North America	35947709
Lindholt et al.	NEJM		Epi I	RCT	Europe (UK excluded)	36027560
Hugosson et al.	NEJM		Epi I	RCT	Europe (UK excluded)	36477032
Gross et al.	NEJM		Epi I	Phase 1/2 study	Multicontinent	36094839
Bramante et al.	NEJM		Epi I	RCT	North America	36070710
Han et al.	NEJM	Smoking [Exposure]	Epi I	RCT	North America	36066078
Nyang'wa et al.	NEJM		Epi I	RCT	Multicontinent	36546625
Hundscheid et al.	NEJM		Epi I	RCT	Europe (UK excluded)	36477458
Devos et al.	NEJM		Epi I	Phase 1/2 study	UK & Other European countries	36449420
Schmoele-Thoma et al.	NEJM		Epi I	Phase 1/2 study	UK	35731653
Russell et al.	NEJM		Epi I	RCT	North America	36170500
Lee et al.	NEJM		Epi I	RCT	North America	36342109

Authors	Journal	Risk factor(s) [role]	Study Type	Study Design[a]	Geographic zone	PMID
Suarez et al.	NEJM	Illicit drugs [Outcome]	Epi O	Cohort	North America	36449419
Hassager et al.	NEJM		Epi I	RCT	Europe (UK excluded)	36342119
Hill et al.	NEJM		Epi I	RCT	North America	36342116
Kelgeri et al.	NEJM		Epi O	Cohort	UK	35830627
Friedman et al.	NEJM		Epi I	Controlled trial	Multicontinent	36036522
Guide et al.	NEJM		Epi I	RCT	North America	36516090
Pellerin et al.	NEJM		Chemical/toxicological, in vivo, in vitro	In vitro/ in vivo	Multicontinent	36516086
Heyland et al.	NEJM		Epi I	RCT	Multicontinent	36082909
Strnad et al.	NEJM		Epi I	Phase 1/2 study	UK & Other European countries	35748699
Werth et al.	NEJM		Epi I	Phase 1/2 study	Multicontinent	35939578
Cowan et al.	NEJM		Epi I	Phase 1/2 study	North America	36546626
Ganetzky et al.	NEJM		Other	Case series	North America	36239646
Anderson et al.	NEJM		Epi I	RCT	North America	36260859
Bloomfield et al.	NEJM		Epi I	RCT	Oceania	36322845
Park et al.	NEJM		Epi I	RCT	Asia	36036496
Cohen et al.	NEJM		Other	Case series	North America	36351280
Burnside et al.	NEJM		Epi I	RCT	Oceania	36069869
Wu et al.	NEJM		Epi I	Phase 1/2 study	North America	35921449
Verweij et al.	NEJM		Chemical/toxicological, in vivo, in vitro	In vitro/ in vivo	North America & UK	35939579
Gould et al.	NEJM		Epi O	Cohort	Oceania	36300974
Halasa et al.	NEJM		Epi O	Case-control study	North America	35731908
Kayentao et al.	NEJM		Epi I	Phase 1/2 study	Africa	36317783
Cowger et al.	NEJM		Epi O	DB analysis	North America	36351262
Boughton et al.	NEJM		Epi I	RCT	UK	36069870
Garcia et al.	NEJM		Epi O	Cohort	North America	36300973
Patel et al.	NEJM		Epi O	Cohort	North America	36053505
Asmis et al.	NEJM		Other	Case series	Europe (UK excluded)	36546627
Kieh et al.	NEJM		Epi I	RCT	Africa	36516078
Tan et al.	NEJM		Epi O	Cohort	Asia	35857701
Chemaitelly et al.	NEJM		Epi O	Cohort	Asia	36322837
Clasen et al.	NEJM		Epi I	RCT	Multicontinent	36214599
Bertaina et al.	NEJM		Epi I	Controlled trial	North America	35704481
Cohen-Stavi et al.	NEJM		Epi O	Cohort	Asia	35767475
Chin et al.	NEJM		Epi O	Cohort	North America	36286260
Epstein et al.	NEJM		Epi I	RCT	North America	36449421
Moreau et al.	NEJM		Epi I	Phase 1/2 study	Multicontinent	35661166
Richardson et al.	NEJM		Epi I	RCT	North America	35660812
Cercek et al.	NEJM		Epi I	Phase 1/2 study	North America	35660797
Tie et al.	NEJM		Epi I	RCT	Oceania	35657320
Papi et al.	NEJM		Epi I	RCT	Multicontinent	35569035
Al-Samkari et al.	NEJM		Epi I	RCT	Multicontinent	35417638
Myhrvold et al.	NEJM		Epi I	RCT	Europe (UK excluded)	35417636
Forde et al.	NEJM		Epi I	RCT	Multicontinent	35403841
Tita et al.	NEJM		Epi I	RCT	North America	35363951
Abdel-Fattah et al.	NEJM		Epi I	RCT	UK	35353961
King et al.	NEJM		Epi I	RCT	Multicontinent	35334197
Cortés et al.	NEJM		Epi I	RCT	Multicontinent	35320644
Hortobagyi et al.	NEJM		Epi I	RCT	Multicontinent	35263519
Leboulleux et al.	NEJM		Epi I	RCT	Europe (UK excluded)	35263518

Authors	Journal	Risk factor(s) [role]	Study Type	Study Design[a]	Geographic zone	PMID
Andrews et al.	NEJM		Epi O	Case-control study	UK	35249272
Maurovich-Horvat et al.	NEJM		Epi I	RCT	UK & Other European countries	35240010
Devereaux et al.	NEJM		Epi O	Cohort	Multicontinent	35235725
Israel et al.	NEJM		Epi I	RCT	North America	35213105
Ruijter et al.	NEJM		Epi I	RCT	Europe (UK excluded)	35196426
Smith et al.	NEJM		Epi I	RCT	Multicontinent	35179323
Hammerman et al.	NEJM		Epi O	Cohort	Asia	35172072
Hammond et al.	NEJM		Epi I	RCT	Multicontinent	35172054
Schmid et al.	NEJM		Epi I	RCT	Multicontinent	35139274
Fink et al.	NEJM		Epi O	Cohort	Africa	35108469
Seifert et al.	NEJM		Review	Review	NA	34986287
John et al.	NEJM		Epi I	RCT	Asia	34986286
Tawbi et al.	NEJM		Epi I	RCT	Multicontinent	34986285
Gottlieb et al.	NEJM		Epi I	RCT	Multicontinent	34937145
Jayk Bernal et al.	NEJM		Epi I	RCT	Multicontinent	34914868
Kanter et al.	NEJM		Epi I	Phase 1/2 study	North America	34898139
Locatelli et al.	NEJM		Epi I	RCT	Multicontinent	34891223
Fearon et al.	NEJM		Epi I	RCT	Multicontinent	34735046
Li et al.	NEJM		Epi I	Phase 1/2 study	Multicontinent	34534430
Strickler et al.	NEJM		Epi I	Phase 1/2 study	Multicontinent	36546651
Priemer et al.	NEJM		Epi O	Case-control study	North America	35675177
Jastreboff et al.	NEJM	Excess body weight [Outcome]	Epi I	RCT	Multicontinent	35658024
Arslanian et al.	NEJM		Epi I	RCT	Multicontinent	35658022
Jänne et al.	NEJM		Epi O	Cohort	North America	35658005
Montgomery et al.	NEJM		Other	Case series	North America	35584156
Creech et al.	NEJM		Epi I	RCT	North America	35544369
Dai et al.	NEJM		Epi I	RCT	Multicontinent	35507481
Liu et al.	NEJM	Excess body weight [Outcome]	Epi I	RCT	Asia	35443107
Magen et al.	NEJM		Epi O	Cohort	Asia	35417631
Squair et al.	NEJM		Other	Case series	Europe (UK excluded)	35388667
Bar-On et al.	NEJM		Epi O	DB analysis	Asia	35381126
Reis et al.	NEJM		Epi I	RCT	Other American countries	35353979
Price et al.	NEJM		Epi O	Case-control study	North America	35353976
Fijen et al.	NEJM		Epi I	Phase 1/2 study	Multicontinent	35294812
Ozelo et al.	NEJM		Epi I	RCT	Multicontinent	35294811
Turkova et al.	NEJM		Epi I	RCT	Multicontinent	35263517
Gee et al.	NEJM		Other	Case series	North America	35235727
Hall et al.	NEJM		Epi O	Cohort	UK	35172051
Doki et al.	NEJM		Epi I	RCT	Multicontinent	35108470
Ware et al.	NEJM		Epi I	RCT	UK & Other European countries	35045227
Makker et al.	NEJM		Epi I	RCT	Multicontinent	35045221
Olson et al.	NEJM		Epi O	Case-control study	North America	35021004
Andrews et al.	NEJM		Epi O	Case-control study	UK	35021002
Tilly et al.	NEJM		Epi I	RCT	Multicontinent	34904799
Bishop et al.	NEJM		Epi I	RCT	Multicontinent	34904798
Lang et al.	NEJM		Epi I	Phase 1/2 study	Multicontinent	35921450

Authors	Journal	Risk factor(s) [role]	Study Type	Study Design[a]	Geographic zone	PMID
Meyhoff et al.	NEJM		Epi I	RCT	UK & Other European countries	35709019
Loayza Mafayle et al.	NEJM		Chemical/toxicological, in vivo, in vitro	In vitro/ in vivo	Other American countries	35704480
Palefsky et al.	NEJM		Epi I	RCT	North America	35704479
Altarawneh et al.	NEJM		Epi O	Case-control study	Asia	35704396
Modi et al.	NEJM		Epi I	RCT	Multicontinent	35665782
Richeldi et al.	NEJM		Epi I	Phase 1/2 study	Multicontinent	35569036
Segal-Maurer et al.	NEJM		Epi I	RCT	Multicontinent	35544387
Hodgson et al.	NEJM		Epi I	RCT	Oceania	35476651
Simões et al.	NEJM		Epi I	Phase 1/2 study	Multicontinent	35476650
Montesinos et al.	NEJM		Epi I	RCT	Multicontinent	35443108
Kuter et al.	NEJM		Epi I	Phase 1/2 study	Multicontinent	35417637
Eckburg et al.	NEJM		Epi I	RCT	Multicontinent	35388666
Devereaux et al.	NEJM		Epi I	RCT	Multicontinent	35363452
Sullivan et al.	NEJM		Epi I	RCT	North America	35353960
Moreira et al.	NEJM		Epi I	RCT	Multicontinent	35320659
Jarvis et al.	NEJM		Epi I	RCT	Africa	35320642
Merrill et al.	NEJM		Epi I	Phase 1/2 study	Multicontinent	35294813
Abu-Raddad et al.	NEJM		Epi O	Cohort	Asia	35263534
Paixao et al.	NEJM		Epi O	Cohort	Other American countries	35196428
Wang et al.	NEJM		Chemical/toxicological, in vivo, in vitro	In vitro/ in vivo	North America	35196427
Madhi et al.	NEJM		Epi O	DB analysis	Africa	35196424
Ahmad et al.	NEJM		Epi I	Phase 1/2 study	UK	35172056
Tewari et al.	NEJM		Epi I	RCT	Multicontinent	35139273
Fernandez et al.	NEJM		Epi I	RCT	UK	35139272
Yoshimura et al.	NEJM		Epi I	RCT	Asia	35138767
Sablerolles et al.	NEJM		Epi I	RCT	Europe (UK excluded)	35045226
Finfer et al.	NEJM		Epi I	RCT	Oceania	35041780
Lin et al.	NEJM		Epi O	Cohort	North America	35020982
Eyre et al.	NEJM		Epi O	Cohort	UK	34986294
Peffault de Latour et al.	NEJM		Epi I	RCT	UK & Other European countries	34986284
Dunkle et al.	NEJM		Epi I	RCT	Multicontinent	34910859
Goyal et al.	NEJM		Other	Case series	North America	34898140
Schummers et al.	NEJM		Epi O	DB analysis	North America	34879191
Gammie et al.	NEJM		Epi I	RCT	Multicontinent	34767705
Tao et al.	NEJM		Epi I	RCT	Asia	36239644
Aggarwal et al.	NEJM		Epi I	RCT	Multicontinent	36198179
Leelarathna et al.	NEJM		Epi I	RCT	UK	36198143
Mailankody et al.	NEJM		Epi I	Phase 1/2 study	North America	36170501
de-Madaria et al.	NEJM		Epi I	RCT	Europe (UK excluded)	36103415
Crowther et al.	NEJM		Epi I	RCT	Oceania	36070709
Furie et al.	NEJM		Epi I	Phase 1/2 study	Multicontinent	36069871
Conradie et al.	NEJM		Epi I	RCT	Multicontinent	36053506
Koo et al.	NEJM		Epi I	RCT	Asia	36053504
Smolen et al.	NEJM		Epi I	RCT	Multicontinent	36001712
Steinman et al.	NEJM		Epi I	RCT	North America	36001711
LeBoff et al.	NEJM		Epi O	Cohort	North America	35939577
Pagano et al.	NEJM		Epi I	Phase 1/2 study	Multicontinent	35921451
Chowdary et al.	NEJM		Epi I	Phase 1/2 study	Multicontinent	35857660

Authors	Journal	Risk factor(s) [role]	Study Type	Study Design[a]	Geographic zone	PMID
Cortes et al.	NEJM		Epi I	RCT	Asia	35857659
Jhaveri et al.	NEJM		Epi I	RCT	North America	35833805
Gutierrez Sanchez et al.	NEJM		Other	Case series	North America	35830653
Ansell et al.	NEJM		Epi I	RCT	Multicontinent	35830649
Wu et al.	NEJM		Epi I	RCT	North America	35830641
Gállego Pérez-Larraya et al.	NEJM		Epi I	Phase 1/2 study	Europe (UK excluded)	35767439
Wang et al.	NEJM		Epi I	RCT	Multicontinent	35657079
Leidner et al.	NEJM		Other	Case series	North America	35648703
Goldberg et al.	NEJM		Epi O	DB analysis	Asia	35613036
Liu et al.	NEJM		Epi O	Cohort	Multicontinent	35613022
Hager et al.	NEJM		Epi I	RCT	Multicontinent	35507508
Levin et al.	NEJM		Epi I	RCT	Multicontinent	35443106
Kamran et al.	NEJM		Epi O	DB analysis	North America	35388674
Jacobs et al.	NEJM	Smoking; Excess body weight [Exposure]	Epi O	Cohort	Multicontinent	35373933
Chamma-Siqueira et al.	NEJM		Epi I	Controlled trial	Other American countries	35353962
Watterberg et al.	NEJM		Epi I	RCT	North America	35320643
Hammitt et al.	NEJM		Epi I	RCT	Multicontinent	35235726
Sadoff et al.	NEJM		Epi I	RCT	Multicontinent	35139271
Atmar et al.	NEJM		Epi I	Phase 1/2 study	North America	35081293
Ytterberg et al.	NEJM		Epi I	RCT	Multicontinent	35081280
Feuerstadt et al.	NEJM		Epi I	RCT	North America	35045228
Brubacher et al.	NEJM	Alcohol; Illicit drugs [Burden]	Epi O	Cross sectional study	North America	35020985
Rosenberg et al.	NEJM		Epi O	Cohort	North America	34942067
Dickerman et al.	NEJM		Epi O	Cohort	North America	34942066
Locke et al.	NEJM		Epi I	RCT	Multicontinent	34891224
Ma et al.	NEJM		Epi O	Cohort	North America	34767706
Beaton et al.	NEJM		Epi I	RCT	Africa	34767321
Walter et al.	NEJM		Epi I	Phase 1/2 study	Multicontinent	34752019

BMJ, British Medical Journal; DB study, database study; Epi I, epidemiological interventional; Epi O, epidemiological observational; JAMA, Journal of the American Medical Association; NA, not available; NEJM, New England Journal of Medicine; RCT, randomized controlled trial; UK, United Kingdom; Vol, volume.
[a] Other: including in vivo and in vitro studies, case series, methodological analyses, qualitative studies, economic analyses, essays and viewpoints.

eTable 3. Characteristics of the 1,128 included articles published in JAMA, BMJ, Lancet, and NEJM in 2022

Characteristics of the included articles	Number (%)
All	1,128 (100.0)
Journal	
JAMA	409 (36.3)
BMJ	239 (21.2)
Lancet	253 (22.4)
NEJM	227 (20.1)
Study type	
Interventional	384 (34.0)
Observational	324 (28.7)
Review	204 (18.1)
Other[a]	216 (19.2)
Country	
UK[b]	136 (12.1)
Northern America	352 (31.2)
European countries other than UK	90 (8.0)
Africa, Asia, Oceania and Other American	103 (9.1)
Africa	17 (1.5)
Asia	54 (4.8)
Oceania	21 (1.9)
Other American countries	11 (1.0)
Multicontinent	174 (15.4)
Not applicable	273 (24.2)
Type of disease[c]	
CVD	173 (15.3)
COVID-19	189 (16.8)
Mental health	128 (11.3)
Cancer	99 (8.8)
Digestive system and metabolic diseases	99 (8.8)
Orthopedic diseases	48 (4.3)
Female reproductive system diseases	52 (4.6)
Respiratory disease	40 (3.5)
STD	56 (5.0)
Other	244 (21.6)

BMJ, British Medical Journal; COVID-19: coronavirus disease 2019; CVD, cardiovascular disease; JAMA, Journal of the American Medical Association; NEJM, New England Journal of Medicine; STD, sexually transmitted diseases and sexuality; UK, United Kingdom.

[a] Other: including in vivo and in vitro studies, case series, methodological analyses, qualitative studies, economic analyses, essays and viewpoints.

[b] Including 11 studies set in UK and other European countries.

[c] Mental health: including also brain diseases/abuse of substances; Digestive system and metabolic diseases: including also kidney, urinary diseases and sedentarism; Orthopedic diseases: including bones/soft tissue damages; Injuries and also dysplasia; Female reproductive system diseases: including also maternal, pregnancy and infant diseases.

eTable 4. Characteristics of the 1,128 included articles published in JAMA, BMJ, Lancet, and NEJM in 2022, according to their focus on any preventable behavioral risk factors, smoking, alcohol use, use of illicit drugs, excess body weight, or physical activity

Characteristics of the included articles	Number	Any preventable behavioral risk factor Number (%)	Smoking[a] Number (%)	Alcohol drinking[a] Number (%)	Use of illicit drugs[a] Number (%)	Excess body weight[a] Number (%)	Physical activity[a] Number (%)
All	1,128	90 (8.0)	31 (2.8)	18 (1.6)	12 (1.1)	43 (3.8)	30 (2.7)
Journal							
JAMA	409	35 (8.6)	10 (2.4)	2 (0.4)	8 (1.9)	13 (3.1)	9 (2.2)
BMJ	239	28 (11.7)	11 (4.6)	6 (2.5)	2 (0.8)	12 (5.0)	14 (5.8)
Lancet	253	20 (7.9)	8 (3.1)	9 (3.5)	0 (0.0)	14 (5.5)	7 (2.7)
NEJM	227	7 (3.1)	2 (0.8)	1 (0.4)	2 (0.8)	4 (1.7)	0 (0.0)
Study type							
Interventional	384	25 (6.5)	6 (1.5)	3 (0.7)	1 (0.2)	10 (2.6)	13 (3.3)
Observational	324	32 (9.9)	13 (4.0)	8 (2.4)	7 (2.1)	16 (4.9)	5 (1.5)
Review	204	25 (12.3)	9 (4.4)	6 (2.9)	0 (0.0)	15 (7.3)	11 (5.3)
Other[b]	216	8 (3.7)	3 (1.3)	1 (0.4)	4 (1.8)	2 (0.9)	1 (0.4)
Country							
UK[c]	136	9 (6.6)	1 (0.7)	1 (0.7)	1 (0.7)	3 (2.2)	3 (2.2)
Northern America	352	38 (10.8)	10 (2.8)	5 (1.4)	10 (2.8)	17 (4.8)	10 (2.8)
European countries other than UK	90	5 (5.6)	3 (3.3)	2 (2.2)	0 (0.0)	1 (1.1)	2 (2.2)
Africa, Asia, Oceania and Other American	103	4 (3.9)	2 (1.9)	3 (2.9)	0 (0.0)	3 (2.9)	2 (1.9)
Africa	17	1 (5.8)	0 (0.0)	1 (5.8)	0 (0.0)	0 (0.0)	0 (0.0)
Asia	54	3 (5.5)	2 (3.7)	2 (3.7)	0 (0.0)	3 (5.5)	2 (3.7)
Oceania	21	0 (0.0)	0 (0.0)	0 (0.0)	0 (0.0)	0 (0.0)	0 (0.0)
Other American countries	11	0 (0.0)	0 (0.0)	0 (0.0)	0 (0.0)	0 (0.0)	0 (0.0)
Multicontinent	174	11 (6.3)	8 (4.5)	2 (1.1)	0 (0.0)	6 (3.4)	4 (2.2)
Not applicable	273	23 (8.4)	7 (2.5)	5 (1.8)	1 (0.3)	13 (4.7)	9 (3.2)
Type of disease[d]							
CVD	173	12 (6.9)	5 (2.8)	1 (0.5)	0 (0.0)	7 (4.0)	7 (4.0)
COVID-19	189	2 (1.1)	0 (0.0)	1 (0.5)	0 (0.0)	1 (0.5)	0 (0.0)
Mental health	128	27 (21.1)	12 (9.3)	6 (4.6)	10 (7.8)	1 (0.7)	2 (1.5)
Cancer	99	7 (7.1)	4 (4.0)	4 (4.0)	0 (0.0)	5 (5.0)	3 (3.0)
Digestive system and metabolic diseases	99	29 (29.3)	3 (3.0)	2 (2.0)	0 (0.0)	24 (24.2)	11 (11.1)
Orthopedic diseases	48	3 (6.3)	0 (0.0)	0 (0.0)	0 (0.0)	0 (0.0)	3 (6.2)
Female reproductive system diseases	52	3 (5.8)	1 (1.9)	1 (1.9)	1 (1.9)	2 (3.8)	1 (1.9)
Respiratory disease	40	3 (7.5)	3 (7.5)	0 (0.0)	0 (0.0)	0 (0.0)	0 (0.0)
STD	56	1 (1.8)	0 (0.0)	0 (0.0)	1 (1.7)	0 (0.0)	0 (0.0)
Other	244	3 (1.2)	3 (1.2)	3 (1.2)	0 (0.0)	3 (1.2)	3 (1.2)

BMJ, British Medical Journal; COVID-19, coronavirus disease 2019; CVD, cardiovascular disease; JAMA, Journal of the American Medical Association; NEJM, New England Journal of Medicine; STD, sexually transmitted diseases and sexuality; UK, United Kingdom.

[a] The risk factor categories (smoking, alcohol use, use of illicit drugs, excess body weight or physical activity) are not mutually exclusive.

[b] Other: including in vivo and in vitro studies, case series, methodological analyses, qualitative studies, economic analyses, essays and viewpoints.

[c] Including 11 studies set in UK and other European countries.

[d] Mental health: including also brain diseases/abuse of substances; Digestive system and metabolic diseases: including also kidney, urinary diseases and sedentarism; Orthopedic diseases: including bones/soft tissue damages; Injuries and also dysplasia; Female reproductive system diseases: including also maternal, pregnancy and infant diseases.

eTable 5. Distribution of papers focusing on selected preventable behavioral risk factors (ie, smoking, alcohol drinking, use of illicit drugs, excess body weight and physical activity) and percentages on the total of 1,128 articles published in JAMA, BMJ, Lancet, and NEJM in 2022, according to the role of the risk factor (outcome, exposure, burden or recommendation) in the paper

		Articles dealing with the…				
	Articles dealing with the behavior as an **outcome** only<br><br>Number (%)	… behavior as an **exposure** only<br><br>Number (%)	… **burden** of the behavior only<br><br>Number (%)	…**recommendations** for the behavior only<br><br>Number (%)	… behavior as **exposure** or its **burden** or its **recommendation**<br><br>Number (%)	Articles dealing with the behavior as an **outcome**, **exposure**, its **burden** or its **recommendation**<br><br>Number (%)
Any behavior	27 (2.4)	30 (2.7)	21 (1.9)	12 (1.0)	63 (5.6%)	90 (8.0)
Smoking	5 (0.4)	13 (1.2)	6 (0.5)	7 (0.6)	26 (2.3%)	31 (2.7)
Alcohol drinking	1 (0.1)	6 (0.5)	6 (0.5)	5 (0.5)	17 (1.5%)	18 (1.6)
Illicit drugs	4 (0.4)	1 (0.1)	7 (0.6)	0 (0.0)	8 (0.7%)	12 (1.1)
Excess body weight	12 (1.1)	16 (1.4)	6 (0.6)	9 (0.7)	31 (2.7%)	43 (3.8)
Physical activity	7 (0.6)	9 (0.8)	4 (0.4)	10 (0.9)	23 (2.1%)	30 (2.7)

BMJ, British Medical Journal; JAMA, Journal of the American Medical Association; NEJM, New England Journal of Medicine.

eTable 6. Accuracy statistics (sensitivity, specificity and Youden's J statistics[a]) referring to the comparison of the classification of papers on selected preventable behavioral risk factors obtained applying search strings in PubMed/MEDLINE versus that obtained manually, considered as the "gold standard", on publications from JAMA, BMJ, the Lancet, and NEJM in 2022

Preventable behavioral risk factors	Statistic		
	**Sensitivity**	**Specificity**	**Youden's J statistic**
Any preventable behavioral risk factor	0.856	0.982	0.837
Smoking	0.871	0.992	0.863
Alcohol drinking	0.722	0.997	0.720
Illicit Drugs	0.667	0.998	0.665
Excess body weight	0.581	0.995	0.577
Physical activity	0.633	0.996	0.630

BMJ, British Medical Journal; JAMA, Journal of the American Medical Association; NEJM, New England Journal of Medicine.

[a] Youden's J statistic computed as: Sensitivity+Specificity-1.

eTable 7. Number of publications (with abstract available) on each preventable behavioral risk factor from all medical literature retrieved using search strings in PubMed/MEDLINE in the years 1993–2022

Year	PubMed (number)						
	**Smoking**	**Alcohol**	**Drugs**	**Excess body weight**	**Physical activity**	**Any behavioral risk factor**	**All publications from PubMed**
2022	24,166	18,730	5,643	29,329	24,098	90,041	1,587,081
2021	24,663	19,045	5,683	29,858	25,480	92,416	1,580,063
2020	23,481	17,597	5,216	27,985	23,387	86,011	1,433,544
2019	20,853	15,922	4,892	24,339	20,705	76,092	1,248,227
2018	20,009	15,464	4,272	23,455	20,006	72,756	1,187,419
2017	19,350	15,040	3,878	22,813	19,110	70,023	1,135,306
2016	19,544	14,591	3,552	22,814	18,670	68,994	1,110,561
2015	19,743	14,152	3,507	22,109	17,890	67,388	1,081,622
2014	18,956	14,008	3,424	21,162	16,893	64,809	1,042,560
2013	18,003	13,270	3,435	19,993	15,460	61,084	991,294
2012	17,173	12,547	3,210	17,486	13,993	56,262	943,919
2011	15,577	11,488	3,069	15,817	12,685	51,222	876,815
2010	14,275	10,485	2,911	14,124	11,226	46,362	806,494
2009	13,377	9,402	2,729	12,620	9,966	42,005	747,395
2008	12,848	9,037	2,919	11,479	9,322	39,849	707,855
2007	11,952	8,654	2,761	9,987	8,293	36,560	662,976
2006	11,280	8,093	2,619	8,450	7,377	33,237	626,183
2005	10,163	7,489	2,311	7,019	6,127	29,071	578,332
2004	8,865	6,524	1,943	5,861	5,403	25,281	518,636
2003	7,796	6,069	1,879	4,760	4,802	22,263	474,863
2002	7,137	5,550	1,823	4,052	4,284	20,195	447,115
2001	6,884	5,361	1,711	3,625	4,080	19,075	426,624
2000	6,451	5,145	1,766	3,259	3,731	18,044	407,571
1999	5,943	4,591	1,564	2,820	3,418	16,270	373,405
1998	5,576	4,382	1,606	2,592	3,002	15,290	361,708
1997	5,102	4,177	1,548	2,262	2,903	14,193	344,401
1996	4,946	4,083	1,562	2,076	2,695	13,631	335,105
1995	4,482	3,850	1,487	1,890	2,565	12,718	324,803
1994	4,374	3,784	1,574	1,649	2,239	12,075	315,518
1993	4,188	3,700	1,532	1,693	2,319	11,900	305,851

eTable 8. Number of publications (with abstract available) on each preventable behavioral risk factor from Lancet and JAMA retrieved using search strings in PubMed/MEDLINE in the years 1993–2022

Year	Lancet (number)							JAMA (number)						
	Smoking	Alcohol	Drugs	Excess body weight	Physical activity	Any behavioral risk factor	All publications from Lancet	Smoking	Alcohol	Drugs	Excess body weight	Physical activity	Any behavioral risk factor	All publications from JAMA
2022	10	8	1	10	3	24	253	14	2	6	9	10	36	409
2021	10	5	1	22	4	34	317	12	4	5	17	9	40	303
2020	10	4	1	10	3	18	223	15	3	10	16	6	37	316
2019	15	12	5	14	4	36	285	12	3	7	10	9	36	273
2018	23	12	2	13	3	39	322	13	9	4	16	8	45	286
2017	24	11	1	14	15	49	348	5	1	3	13	10	28	252
2016	19	15	4	11	13	51	392	13	1	1	12	8	27	226
2015	22	10	1	20	6	56	530	9	1	2	9	8	25	217
2014	17	6	1	14	2	31	350	11	5	0	11	5	29	243
2013	22	7	2	12	3	40	336	11	5	2	15	7	34	237
2012	20	9	1	10	11	41	344	15	3	3	23	9	39	234
2011	22	6	4	13	8	38	310	13	3	1	7	5	25	221
2010	12	9	8	9	2	33	281	9	6	1	10	7	25	233
2009	15	11	3	8	4	32	288	9	5	3	7	7	24	237
2008	16	3	4	6	3	28	297	7	4	5	12	8	29	228
2007	18	8	2	6	2	32	294	18	4	0	15	9	35	230
2006	12	6	1	10	6	27	293	26	5	1	11	12	37	262
2005	21	5	2	18	6	41	351	21	10	2	17	8	43	319
2004	18	7	2	7	3	29	407	25	11	1	13	16	48	337
2003	15	5	3	6	3	28	489	22	14	2	18	12	50	335
2002	15	9	3	9	4	33	522	22	10	3	11	15	48	344
2001	22	6	3	10	4	40	567	18	14	4	11	12	45	335
2000	13	5	1	6	5	29	722	26	18	6	3	6	47	330
1999	10	3	5	2	4	24	635	30	9	1	21	16	55	328
1998	12	6	4	4	4	27	360	27	17	10	4	6	51	373
1997	15	5	2	3	1	24	332	24	22	2	5	6	47	337
1996	19	10	3	4	3	33	356	27	17	4	6	14	51	309
1995	9	6	1	2	0	17	403	28	9	2	5	11	45	342
1994	16	1	1	4	2	24	435	35	16	5	4	7	52	343

Year	Lancet (number)							JAMA (number)						
	**Smoking**	**Alcohol**	**Drugs**	**Excess body weight**	**Physical activity**	**Any behavioral risk factor**	**All publications from Lancet**	**Smoking**	**Alcohol**	**Drugs**	**Excess body weight**	**Physical activity**	**Any behavioral risk factor**	**All publications from JAMA**
1993	12	7	2	4	8	25	429	26	15	5	4	7	41	302

eTable 9. Number of publications (with abstract available) on each preventable behavioral risk factor from the BMJ and the New England Journal of Medicine retrieved using search strings in PubMed/MEDLINE in the years 1993–2022

Year	BMJ (number)							NEJM (number)						
	Smoking	Alcohol	Drugs	Excess body weight	Physical activity	Any behavioral risk factor	All publications from BMJ	Smoking	Alcohol	Drugs	Excess body weight	Physical activity	Any behavioral risk factor	All publications from NEJM
2022	10	5	1	6	10	27	239	2	1	2	5	0	9	227
2021	7	9	3	7	3	20	273	0	1	3	4	0	8	252
2020	12	6	1	8	7	28	264	6	1	0	6	2	13	255
2019	18	5	1	11	10	37	287	4	0	1	3	0	8	241
2018	16	8	2	20	13	39	244	5	0	0	5	1	11	237
2017	16	10	4	8	6	37	210	3	0	1	7	2	11	226
2016	14	5	2	6	10	25	212	4	3	2	9	5	18	244
2015	21	10	4	2	8	32	234	8	2	2	8	4	19	296
2014	14	7	1	12	11	30	217	5	0	1	7	1	13	276
2013	23	9	0	14	7	40	227	8	1	0	4	6	16	257
2012	27	11	3	12	16	55	273	6	0	0	9	3	17	254
2011	10	8	1	9	9	28	240	9	2	0	11	8	23	240
2010	18	11	3	11	11	39	250	7	2	1	8	7	20	236
2009	29	7	2	12	13	48	268	9	2	1	8	1	18	237
2008	21	16	4	12	10	51	539	12	4	1	8	2	21	231
2007	13	11	2	13	11	46	716	6	1	0	6	2	13	221
2006	13	8	4	6	3	31	401	6	1	1	6	2	11	215
2005	23	10	3	7	2	37	423	8	2	0	4	8	18	225
2004	21	8	2	7	3	31	427	4	1	0	6	5	13	206
2003	16	8	2	4	5	31	357	7	3	2	7	5	23	217
2002	9	6	2	3	2	20	232	15	5	1	4	7	26	212
2001	20	3	2	9	4	31	192	9	9	1	3	3	18	213
2000	22	3	0	5	0	26	232	11	2	1	4	7	19	229
1999	25	14	0	5	7	38	253	8	11	2	2	4	22	227
1998	23	10	4	1	6	32	245	11	3	0	6	6	21	235
1997	32	17	1	7	4	48	304	10	4	0	2	2	16	225
1996	41	11	4	6	5	53	363	13	7	3	3	3	27	236

Year	BMJ (number)							NEJM (number)						
	Smoking	Alcohol	Drugs	Excess body weight	Physical activity	Any behavioral risk factor	All publications from BMJ	Smoking	Alcohol	Drugs	Excess body weight	Physical activity	Any behavioral risk factor	All publications from NEJM
1995	36	13	4	8	10	50	363	14	6	4	7	3	28	242
1994	39	16	4	2	6	47	383	8	3	3	3	4	18	249
1993	39	12	3	7	7	53	394	15	6	2	5	8	28	252

BMJ, British Medical Journal; NEJM, New England Journal of Medicine.

eFigure 1. Joinpoint analyses[a] for proportion of publications on preventable behavioral risk factors in JAMA, BMJ, the Lancet, and the NEJM, in the years 1993–2022

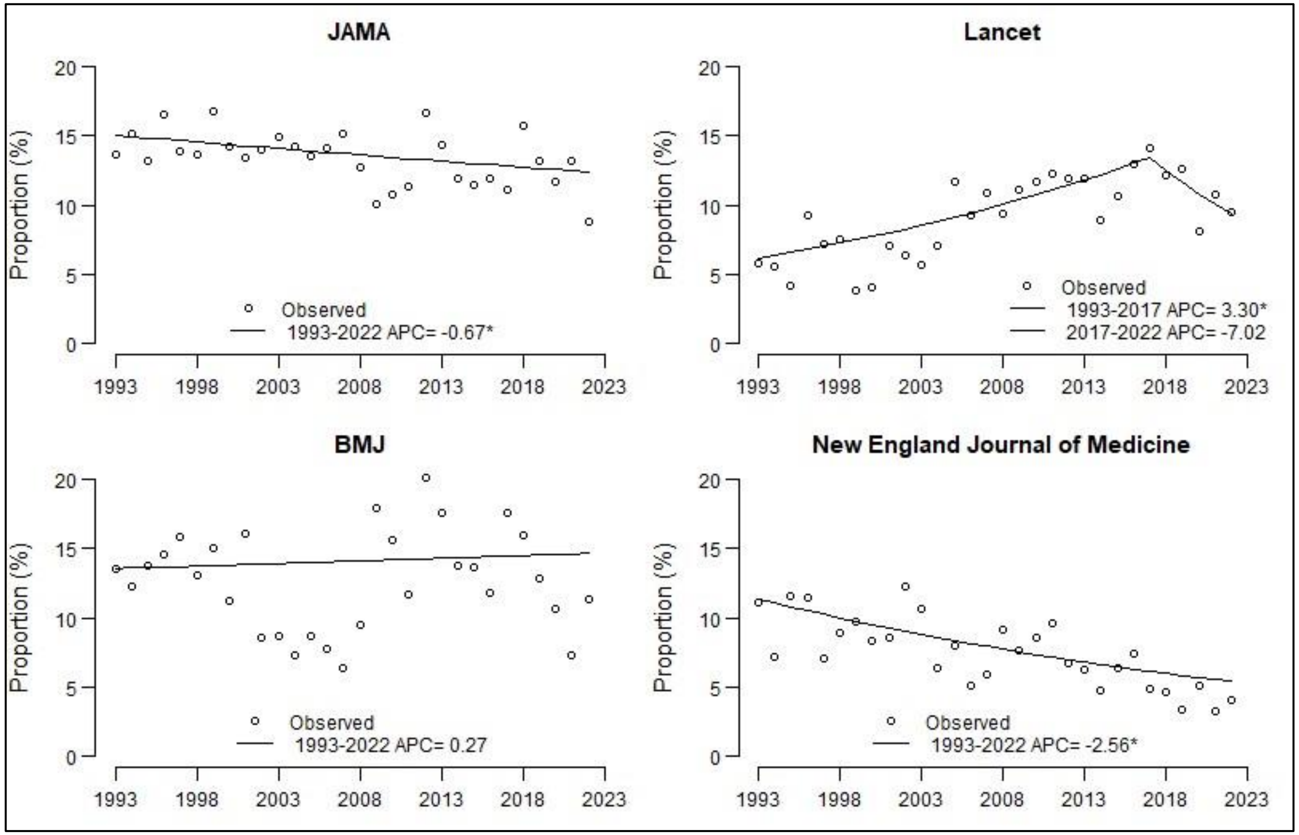

APC, annual percent change; BMJ, British Medical Journal; JAMA, Journal of the American Medical Association; NEJM, New England Journal of Medicine.

[a] Data were obtained from PubMed online database. Joinpoint regression models were fitted allowing for up to 6 joinpoints.

* Indicates that the APC is significantly different from zero at the alpha=0.05 level.
